# A follow up study of cycle threshold values of SARS-CoV-2 in Hunan Province, China

**DOI:** 10.1016/j.idm.2023.01.004

**Published:** 2023-01-20

**Authors:** Guzainuer Abudurusuli, Kaiwei Luo, Xiaohao Guo, Zeyu Zhao, Yichao Guo, Buasiyamu Abudunaibi, Shiting Yang, Hongjie Wei, Shanlu zhao, Zhihui Dai, Qianlai Sun, Hao Yang, Shixiong Hu, Tianmu Chen

**Affiliations:** a State Key Laboratory of Molecular Vaccinology and Molecular Diagnostics, School of Public Health, Xiamen University, Xiamen City, Fujian Province, People's Republic of China; b Hunan Provincial Center for Disease Control and Prevention, Changsha City, 410001, Hunan Province, People's Republic of China

**Keywords:** SARS-COV-2, CT values, Delta variant, Influencing factors

## Abstract

Since the epidemic of the severe acute respiratory syndrome coronavirus 2 (SARS-COV-2), many governments have used reverse transcription polymerase chain reaction (RT-PCR) to detect the virus. However, there are fewer measures of CT values information based on RT-PCR results, and the relationship between CT values and factors from consecutive tests is not clear enough. So in this study, we analyzed the connection between CT values and the factors based on cohort data from Delta variant of SARS-CoV-2 in Hunan Province. Previous studies have showed that the mean age of the cases was 33.34 years (±18.72 years), with a female predominance (55.03%, n = 71), and the greatest proportion of clinical symptoms were of the common type (60.47%, n = 78). There were statistical differences between the N and ORF1ab genes in the CT values for the cases. Based on the analysis of the association between CT values and the factors, the lowest CT values were obtained for the unvaccinated, older and clinically symptomatic group at 3–10 days, the maximum peak of viral load occurred. Therefore, it is recommended to use patient information to focus on older, clinically symptomatic, unvaccinated patients and to intervene promptly upon admission.

## 1 Introduction

The severe acute respiratory syndrome coronavirus2 (SARS-COV-2) has swept the globe since the discovery of the novel coronavirus in December 2019, and the SARS-COV-2 epidemic has been ongoing for more than two years (JIANHUI, 2020). With more than 340 million confirmed cases of SARS-COV-2 reported worldwide as of May 2022, the virus has severely impacted economies worldwide, crowding out other medical visits. The virus has severely impacted economies worldwide, crowding out other medical visits and placing an enormous burden on healthcare workers, who are in a very exhausted state (KEVADIYA et al., 2021; KHANDIA et al., 2022). To date, reverse transcriptase polymerase chain reaction (RT-PCR) testing has only been used for routine detection of viruses in respiratory secretions (TSCHOELLITSCH et al., 2021). Many public health initiatives are rarely based on CT value information, and the association between CT and factors of disease is not well understood.

SARS-COV-2 infection has a wide range of clinical manifestations, from asymptomatic to symptomatic, including fever, dyspnea, fatigue, dry cough, myalgia, lymphopenia and imaging findings of pneumonia, and in severe and critical cases, patients can develop acute respiratory distress syndrome (ARDS), acute respiratory failure, other serious complications and even death (CHEN et al., 2020) (HUANG et al., 2020). Although most asymptomatic patients do not have obvious clinical symptoms, which makes prevention and control efforts more difficult. Currently, some Chinese cities use the commonly available RT-PCR test, which can detect asymptomatic patients in a timely manner, but the information on CT values of patients was not further analyzed and utilized to provide an evidence for public health strategies.

RT-PCR detects viral nucleic acids in the throat, nasopharynx, and nasal airways of patients with suspected SARS-COV-2 during the acute phase of infection, and RT-PCR tests provide semiquantitative results in the form of cyclic threshold CT values (HAY et al., 2021). CT values indicate the number of amplification cycles required for target genes to exceed threshold levels, and these values are logarithmically inversely proportional to viral load; therefore, many studies assess viral load by CT values (WANG et al., 2020a), with lower CT values associated with higher viral load (RAO et al., 2020). Some studies have shown that lower CT values correlate with disease severity (RABAAN et al., 2021). Most published articles have focused on the difference between severe and non-severe SARS-COV-2 cases (LUCAS et al., 2020) (ZHAO et al., 2020) or on the differences in detection rates between nasal and pharyngeal swabs (WANG et al., 2020b). According to one study, clinically symptomatic patients had a 60-fold higher viral load and longer time for viral elimination than clinically minimally symptomatic patients, suggesting that viral load plays a role not only in diagnosis but also in prognosis and transmission (BULLARD et al., 2020). A previous study from Iran showed that of all key factors, sex and age had the greatest impact on SARS-COV-2 symptoms (AZIZMOHAMMAD LOOHA et al., 2022) (FALAHI & KENARKOOHI, 2021). Assessing the sex specificity of different age groups is important in determining the impact of SARS-COV-2 infection, with important implications for health changes. Vaccination is actively promoted in many countries according to the WHO recommendations. However, the effectiveness of vaccination is difficult to assess directly as studies showing that vaccines can reduce new infections and prevent transmission and the need for intensive care units (ALTMANN & BOYTON, 2022). This study assesses whether vaccination is effective by comparing CTs between different vaccination status groups. CT values can also help clinical practitioners determine the need for isolation and quarantine, predict disease severity, and help public health workers allocate medical resources efficiently.

The purpose of this study was to use data from isolated patients to analyze the trend of CT values over time and explore its relationship with influencing factors, as well as observing whether the CT value information can provide valuable information to medical practitioners so that they can make more targeted measures.

## 2 Method

### 2.1 Data collection

We collected data of SARS-COV-2 cases and close contacts from July 22 to August 14, 2021 in Hunan Province. Data included patient sex, age, date of exposure, date of onset, case type, vaccination status, clinical severity, source of infection, and CT values. Data for this study were obtained from the Hunan Provincial Center for Disease Control and Prevention, an effort to prevent and control the SARS-COV-2 outbreak in accordance with the requirements of the public health policy of the National Health Commission of the People's Republic of China. According to the Infectious Disease Prevention and Control Law, cases should provide relevant information truthfully, so no individual informed consent was required, and the analyzed data set was constructed anonymously.

### 2.2 Study design

In this study, we created a retrospective cohort of 129 cases, based on inclusion-exclusion criteria: (1). Complete basic information (age, residence, date of onset, CT value, etc.), positive nucleic acid test; and exclusion criteria: (1). Insufficient information on CT value (n = 31). Ninety-eight cases were included according to the above criteria, with the first case entering the cohort on 22 July 2021 and the last case entering the cohort on 14 August., for all cases, CT values were collected for their daily tests, and collection sites included nasal and pharyngeal swabs, and two target genes were simultaneously amplified and tested during real-time RT-PCR assays, including open reading frame 1 ab (ORF1ab) and nucleocapsid protein. Our study included four aspects, namely analysis of the epidemiological characteristics of the outbreak, analysis of the consecutive observed CT values for each case, analysis of the overall trend of CT values for all cases from the date of exposure, and analysis of the relationship between age, sex, collection site, exposure date, clinical severity and CT values.

### 2.3 Data analysis

In this study, data were collated and analyzed using MATLAB R2021b. MATLAB was applied to analyze the relationship between age, disease symptoms, date of exposure, site of collection, sex, vaccination status and N gene and ORF1ab gene as well as data visualization. CT values were fitted using the function $y=b1-b2x^{-0.5}+b3x^{-1}$,and statistically analyzed by SPSS 24.0, with t-tests for two-group comparisons and ANOVA or Kruskal-Wallis tests for multiple group comparisons, and in all analyses, means and standard deviations (SD) for all variables are given as descriptive statistics, and categorical variables were reported as frequencies and percentages. All tests were two-tailed and *P* < 0.05 was considered significantly different.

## 3 Result

### 3.1 Epidemiological characteristics

**Fig. 1:**
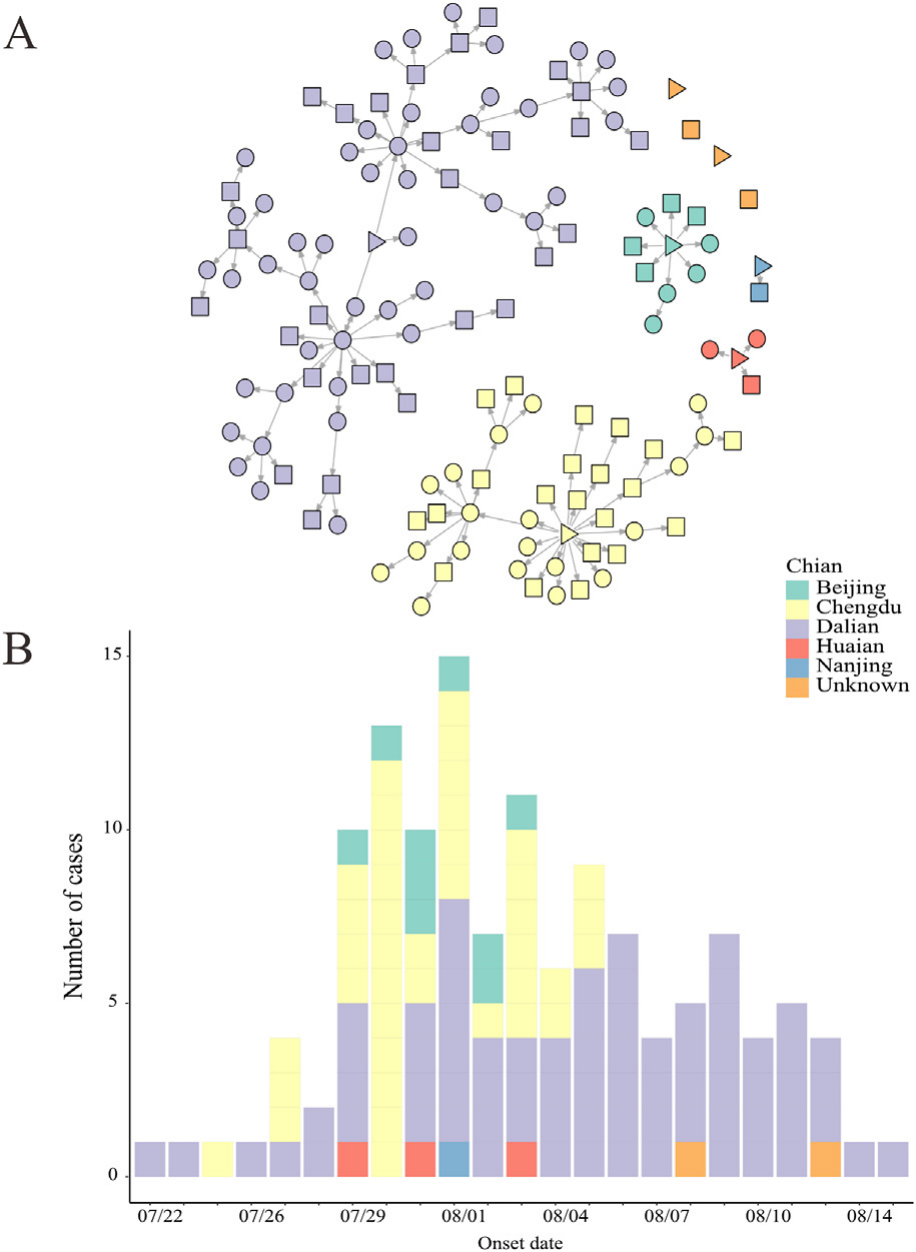
Epidemic transmission chains and epidemic curves. A: Chain of transmission of the epidemic in Hunan Province; B: Epidemiological curve of the epidemic in Hunan Province.

The study included 129 patients diagnosed with SARS-COV-2, with a mean age of 33.34 years (±18.72 years) for individuals, a female predominance (55.03%, n = 71), and the highest proportion of clinical symptoms of the common type (60.47%, n = 78). The outbreak was directly associated with Nanjing Lukou Airport, with spillover involving Beijing, Dalian, Nanjing, Chengdu, and Huai'an. Dalian had the highest number of cases directly associated with it and the longest duration of the outbreak, followed by Chengdu with the highest number of new cases in a single day (Fig. 1).

### 3.2 CT values

**Fig. 2:**
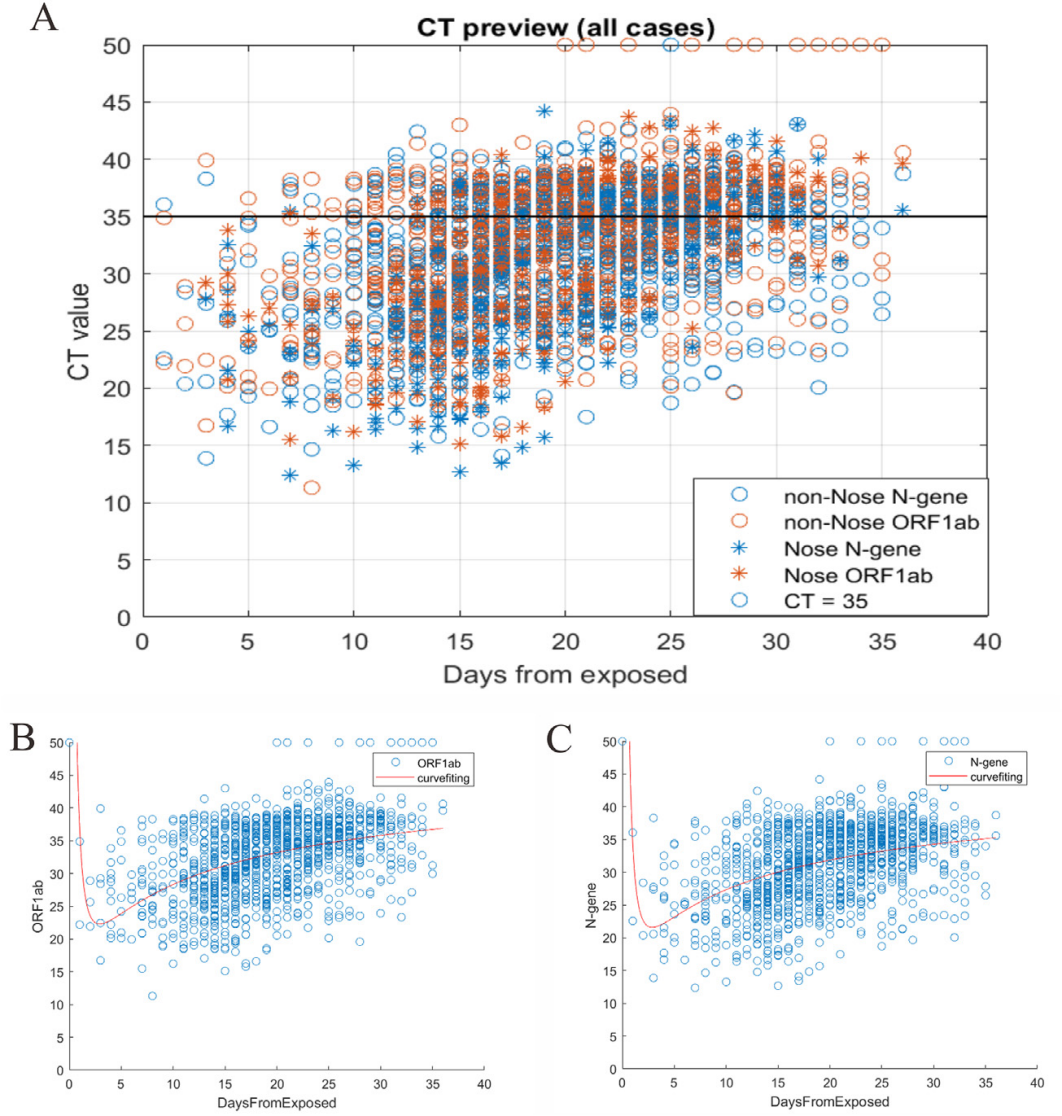
Trend in CT values starting with days of exposure. A: Trend in CT values from days of exposure for all cases; B: Fitting curve of N-gene; C: Fitting curve of ORF1ab gene.

**Fig. 3:**
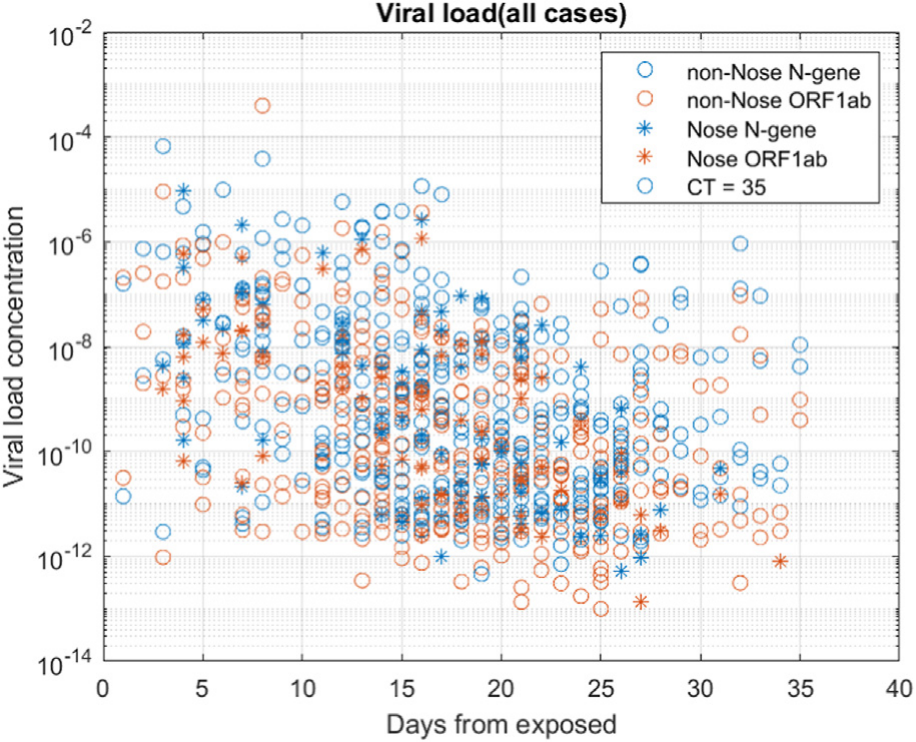
Trend in viral load with days of exposure. Trend in viral load with onset of exposure days for all cases.

A total of 129 cases were included in this study, and according to the inclusion-exclusion criteria, cases without CT values were excluded, leaving 98 cases, and the daily CT values of these 98 cases were visualized (see Appendix for details). In order to better observe the trend of CT values, the CT values of 98 cases were analyzed versus the days from exposure, and after fitting the function, $y=50.13-94.65x^{-0.5}+80.7x^{-1}$ was obtained (All of three fitted parameters significantly differ from 0, with their *p* Values lesser than 0.01), which showed that the CT values were larger at the time of initial exposure, decreasing and reaching a minimum value at 3–10 days. The CT values gradually increased after exposure dates were greater than 10 days, showing a more pronounced upward trend, and most cases having CT values close to 40 after 25 days (Fig. 2). The CT value was inversely proportional to the viral load, so the relationship between the viral load and the number of days after exposure was obtained, the viral load was low at the beginning of the exposure, with an increase in the number of days after exposure, the viral load showed an increasing trend, with the viral load at the highest value at 3–10 days, after 10 days the viral load started to decline, showing a significant downward trend (Fig. 3).

### 3.3 CT values with influencing factors

**Fig. 4:**
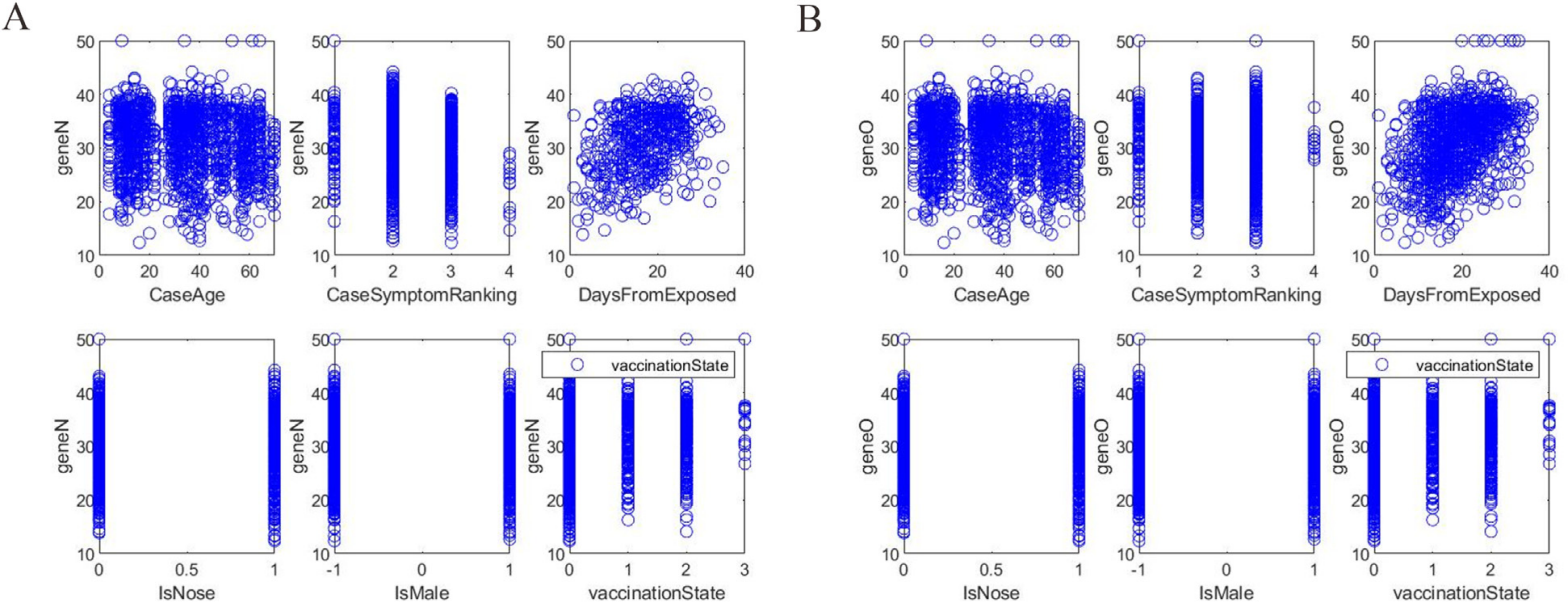
Association between CT value genes with various factors. A: Association between N-gene with various influencing factors; B: Association between ORF1ab gene with various influencing factors.

The CT values included the ORF1ab gene and the N gene (N-gene), and analysis of these two genes made into a paired *t*-test yielded a mean (± standard deviation [SD]) of 31.02 ± 6.00 for the N gene and 32.36 ± 6.08 for the ORF1ab gene, with a *P* < 0.05 for comparison between the two groups, indicating a difference in the CT values of these two genes in those 98 cases (Table 1). Therefore, the relationship between CT values and influencing factors was analyzed to distinguish the ORF1ab gene and N gene and influencing factors. We found that only the days from exposure had a clear linear relationship with CT values (Fig. 4), and there was no further relationship between the remaining factors and the CT values, which were analyzed on a step-by-step basis.

**Table 1:** Statistical analysis of the N-gene and ORF1ab gene.

variable	N	Mean ± std	*p*
N gene	1470	31.02 ± 6.00	<0.05
ORF1ab	1445	32.36 ± 6.08

**Fig. 5:**
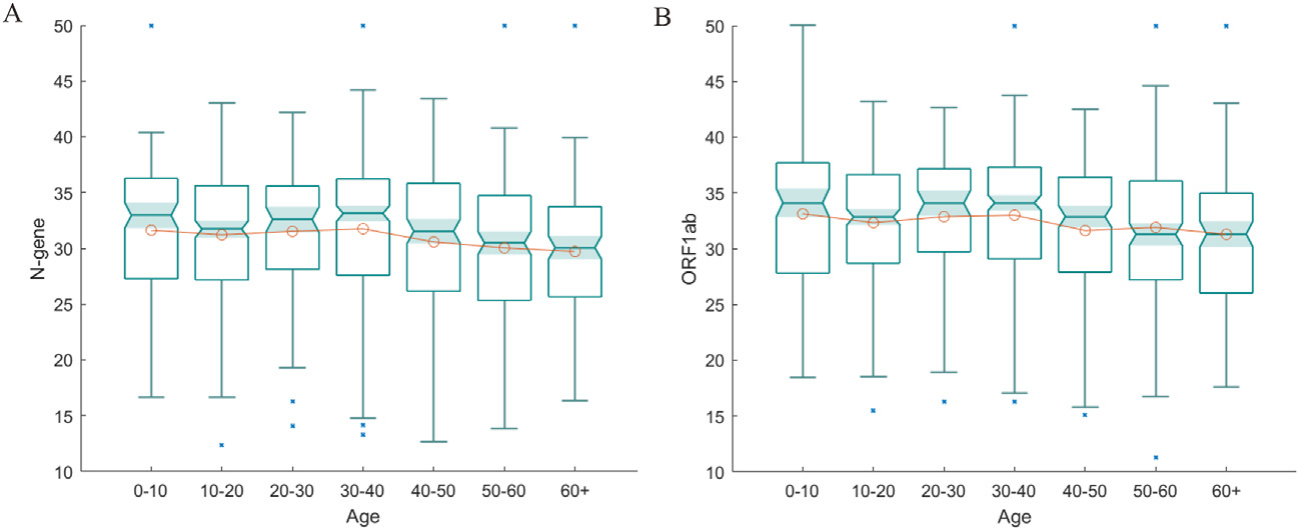
Association between CT value genes with age. A: Association between N-gene with age; B: Association between ORF1ab gene with age.

**Fig. 6:**
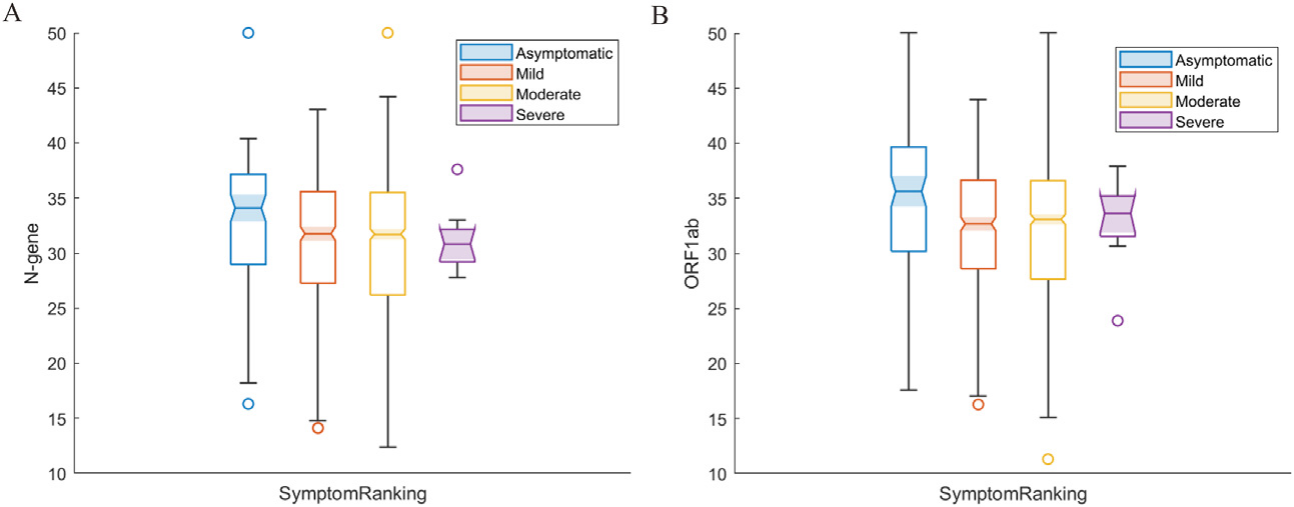
Association between CT value genes with Clinical Symptom Class. A: Association between N-gene with Clinical Symptom Class; B: Association between ORF1ab gene with clinical symptom class Note: One can conclude the CT value in one group is greater than the other group with 95% confidence if the notches of boxplots do not overlaps, The sample size for the severe group is small, so the notches is a bit large.

**Fig. 7:**
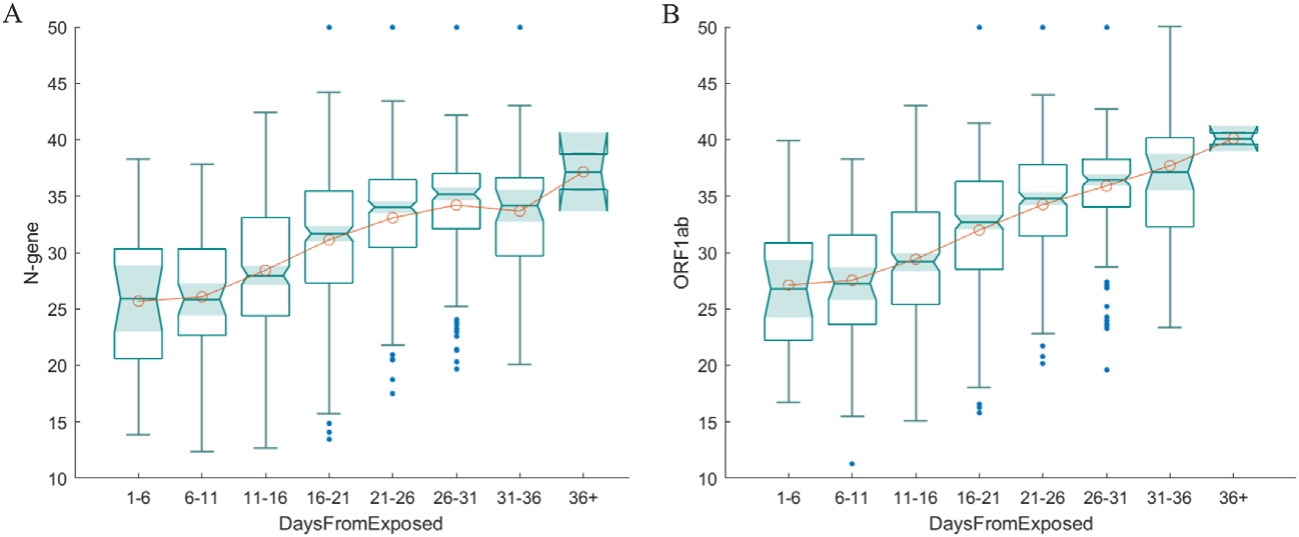
Association between CT value genes with days from exposed. A: Association between N-gene with days from exposed; B: Association between ORF1ab gene with days from exposed.

**Fig. 8:**
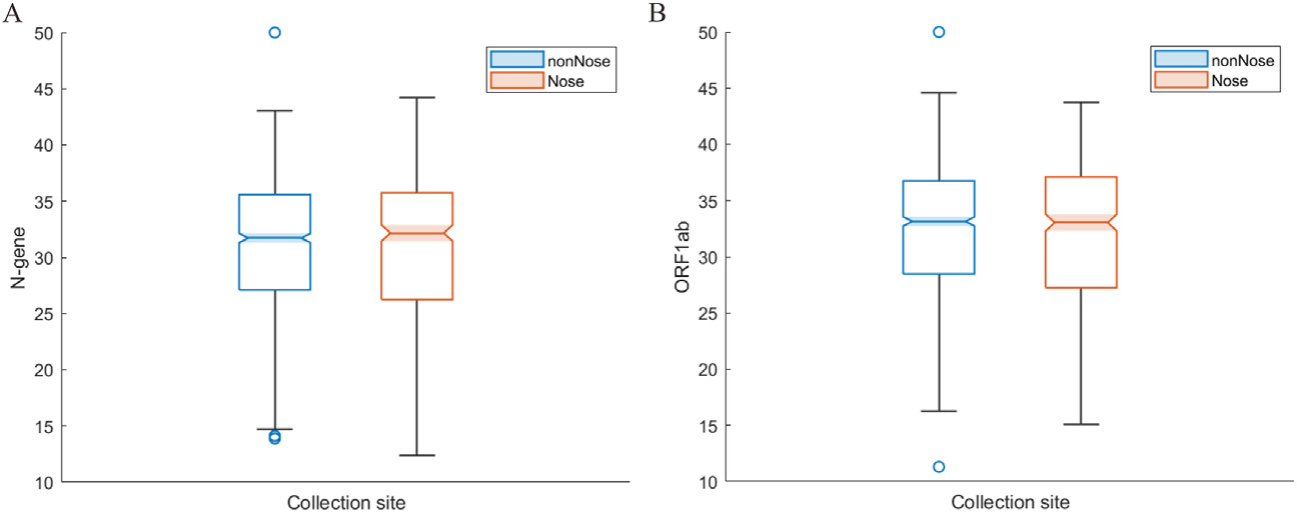
Association between CT value genes with collection site. A: Association between N-gene with collection site; B: Association between ORF1ab gene with collection site.

**Fig. 9:**
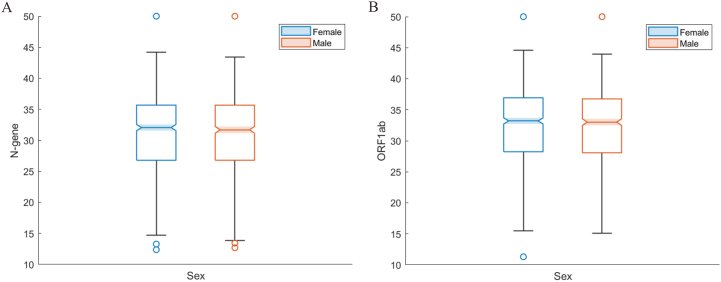
Association between CT value genes with sex. A: Association between N-gene with sex; B: Association between ORF1ab gene with sex.

**Fig. 10:**
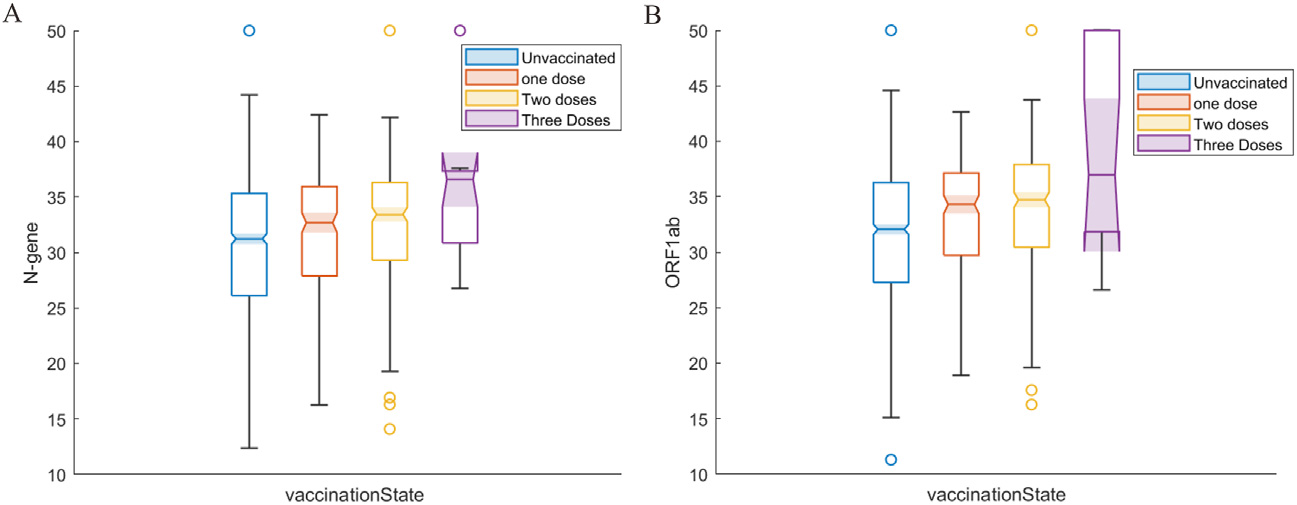
Association between CT value genes with vaccination status. A: Association between N-gene with vaccination status; B: Association between ORF1ab gene with vaccination status Note: The sample size for the three doses group is small, and the notches is a bit large.

The relationship between age and the N gene and ORF1ab gene was first analyzed by dividing ages into seven left-closed, right-open intervals with an interval of 10 and plotting box plots with 95% intervals. There were not significant differences among age groups on the N gene and ORF1ab gene trends, both with the smallest CT values in the 60+ interval, followed by larger CT value in the 10–20 years interval, and also in the 30–40 years interval and the 0–10 years interval (Fig. 5), with statistical analysis yielded significant differences between the age intervals. The clinical symptoms were asymptomatic, mild, moderate and severe patients, and these four clinical symptoms were compared with the N gene and ORF1ab gene, in the N gene, the value was decreasing with the increase of symptoms severity, with the result obtained by statistical analysis was *P* < 0.05, and the result obtained by continued analysis was that there was a statistical difference between the mild and common type groups and the asymptomatic infection group. As for the ORF1ab gene, there was no significant trend between the four groups of clinical symptoms and the ORF1ab gene, the light group had the lowest ORF1ab gene, followed by the common type, and the group with the highest ORF1ab gene was the asymptomatic infected (Fig. 6). To facilitate a comparison of the relationship between days after exposure and CT values, the days after exposure were divided into eight intervals with the left closed and right open. As the number of days after exposure increased, N gene and ORF1ab showed an upward trend, with the lowest CT values at 0–5 days post-exposure, with a median of 25.54 for N gene and a minimum of 13.88, and a median of 26.69 for ORF1ab gene and a with a minimum value of 16.74, followed by the lowest CT values at 5–10 days, while the N gene and ORF1ab gene were already close to 40 at 35+ days after exposure (Fig. 7). Based on the data collected, the collection sites included both nasal and non-nasal swabs and there was no statistical difference between nasal and non-nasal swabs, either in the N gene or in the ORF1ab gene, with a mean of 31.14 ± 5.78 in the N gene and 30.73 ± 6.5 in the non-nasal swab; in the ORF1ab gene the mean nasal swab was 31.74 ± 6.14 and the non-nasal swab was 32.61 ± 6.04 (Fig. 8). There were also no statistically significant differences in the N gene and ORF1ab gene between the sexes (Fig. 9). Vaccination status was unvaccinated, one dose, two doses and three doses, and the N gene and ORF1ab gene were increased with increasing doses of vaccine, and there was a statistically significant difference between the unvaccinated group and the one and two doses (*P* < 0.05) (Fig. 10).

## 4 Discussion

This study analyzed the relationship between CT values and influencing factors in hospitalized patients. To the best of our knowledge, the prominent result of this article is the use of CT values measured continuously during the patient's hospitalization, the analysis of their relationship with influencing factors, the variation of CT values within different factors, and the suggestion in this study that CT values can represent viral load. Several results showed that there was a minimum value of CT values, and a maximum peak in viral load, in the 3–10 days of exposure, non-vaccinated, older, clinically symptomatic group.

Polymerase chain reaction (RT-PCR) is now a common tool for detecting positive infections of SARS-CoV-2, which a semi-quantitative measurement, and standard RT-PCR assays can be performed for up to 40 thermal cycles and are characterized by rapid detection, high sensitivity, and high characterization (YANG et al., 2020) and can therefore be used for early diagnosis (TAHAMTAN & ARDEBILI, 2020). A low CT value indicates a high concentration of genetic material and is thought to be associated with a high risk of infection. CT values from RT-PCR can estimate the viral load in samples from infected individuals (WALSH et al., 2020) (TSUKAGOSHI et al., 2021). According to available studies, the lower the CT value, the higher the viral load. Many studies have analyzed factors affecting CT values or trends in CT values, but unfortunately, most studies have not used continuous patient measurements of CT values and associations with influencing factors, and a large number of studies have focused only on factors affecting CT values (WARD et al., 2021) and have not examined trends in continuous CT values versus factors. The present study analyzed the trend in CT values with an increasing number of days since exposure and found that there was a minimum value of CT at 3–10 days of exposure and then a gradual increase, a finding that could be important in the development of control and treatment duration.

In contrast to other studies, the present study found an association between CT values and disease severity, contrary to the findings of a previous study that nevertheless showed a lack of clarity in the relationship between viral load and disease severity (PAWAR et al., 2022). However, there are also studies, for example, Liu et al. who showed that viral load was higher in severe cases than in mild cases (LIU et al., 2020). Magleby et al. reported an increased risk of latency and death with increasing viral load (CAO et al., 2020). Although the results of these studies are consistent with our findings, these studies used single test results and did not perform serial nucleic acid testing on patients to observe trends in viral load or CT values, whereas the present study used serial CT values for each patient, allowing better analysis of their trends to the days from exposure, and patients with clinical symptoms in the present study obtained statistically lower CT values compared with asymptomatic patients. Older age is a major risk factor for severe SARS-COV-2 (MCKEIGUE et al., 2020). There was a significant connection between age and CT in our study, with a progressive increase in CT values with increasing age, which is consistent with the findings of a Swiss study (LADOY et al., 2021). In this study, when analyzing the days with the lowest CT values, we found that there was a minimum value of CT at 3–10 days after exposure, the highest peak viral load. This is consistent with the results of an article published in The Lancet Infectious Diseases (PAN et al., 2020). The pharyngeal swab test is preferred for most PT-PCR testing in China, but the data we collected included both nasal and pharyngeal swabs and the statistical analysis of CT values at these two sites was not different, unlike other findings that obtained that nasal swabs are more sensitive and have higher viral loads than pharyngeal swabs (ZOU et al., 2020) (XIAO et al., 2020) (WANG et al., 2020b). In our study, there were no differences in CT values between men and women, which is consistent with the results of a multicenter cross-sectional study (ABDULRAHMAN et al., 2021). These findings may have public health implications as public health agencies can categorize potentially seriously ill patients after diagnosis at the time of an outbreak in order to focus medical surveillance on high-risk patients at an early stage at low cost, allocate medical resources efficiently, and help physicians better understand the correlation between CT values and age, vaccines, clinical symptoms, and days of exposure in early intervention and clinical care, as well as prioritize and focus patient care.

### 4.1 Limitations

Although this study used statistical analysis to analyze statistical differences in continuous CT values between factors, the number of patients may lead to bias in the analysis of differences between CT values, and the fact that there were few severe cases and no deaths in this study may have affected the differences in CT values between clinical symptom classes.

## 5 Conclusion

CT values differed statistically between clinical symptoms, vaccination status, age, and days after exposure, i.e. there was a minimum CT value, i.e. a maximum peak in viral load, at 3–10 days of exposure, at an older age, when clinical symptoms were evident and when no vaccination was given.

## Ethics approval and consent to participate

This effort of disease control was part of the CDC's routine responsibility in Hunan Province, China. Therefore, institutional review and informed consent were not required for this study. All data analyzedanalyzed were anonymized.

## Consent for publication

Not applicable.

## Author contributions

GA, ZYZ, XHG, SXH and TMC conceived and designed the study; KWL, SXH, SLZ, ZHZ, QLS and HY provided original data. GA, XHG, TNC and ZYZ conducted the analysis and the framework of the study. GA, XHG, KWL, STY, YCG, SXH, TMC, ZYZ, SLZ, ZHZ, QLS and HY wrote the first draft of the manuscript. GA, BA, ZYZ, YCG, STY, HJW, SXH, KWL and XHG improved this research and edited the manuscript. GXH wrote MATLAB codes; All authors contributed to revising subsequent versions of the manuscript. All authors read and approved the final manuscript.

## Funding

This study was partly supported by The 10.13039/501100012166National Key Research and Development Program of China (2021YFC2301604), the 10.13039/100000865Bill & Melinda Gates Foundation (INV-005834), Hunan Provincial Innovative Construction Special Fund: Emergency response to COVID-19 outbreak (No. 2020SK3012), 10.13039/501100005150Chinese Academy of Medical Sciences Coronavirus Disease 2019 Science and Technology Research Project in 2020 (No. 2020HY320003) and Hunan Workstation for Emerging Infectious Disease Control and Prevention, 10.13039/501100005150Chinese Academy of Medical Sciences.

## Declaration of competing interest

The authors declare that they have no known competing financial interests or personal relationships that could have appeared to influence the work reported in this paper.
