# Supplementary material for: A follow up study of cycle threshold values of SARS-CoV-2 in Hunan Province, China
Source: Infect Dis Model. 2023 Jan 20;8(1):203–11. doi: 10.1016/j.idm.2023.01.004 (PMC9851914; doi:10.1016/j.idm.2023.01.004)
Supplement: Multimedia component 1 [file mmc1.doc]

**Supplementary Materials: Figures of daily CT values of 98 cases**


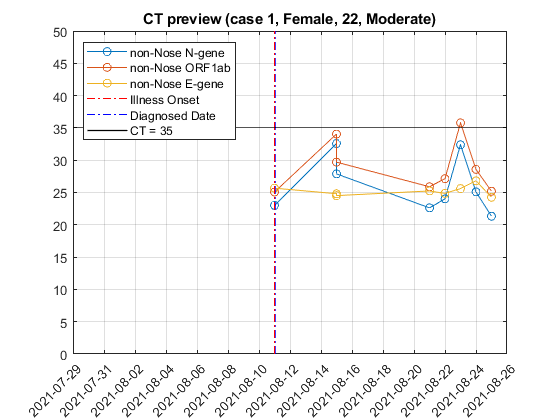


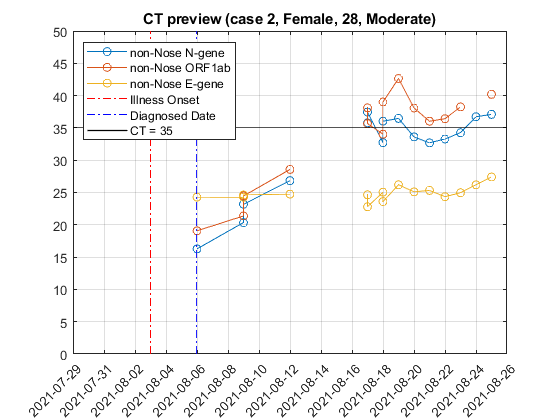


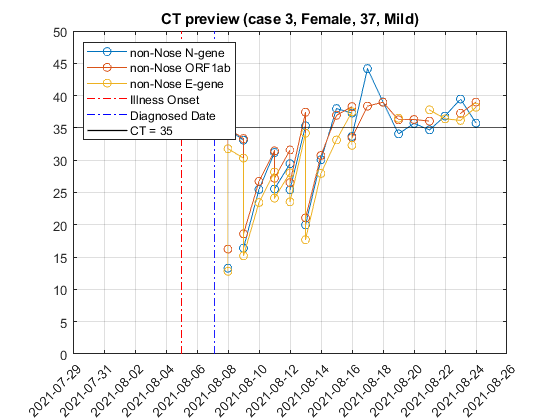


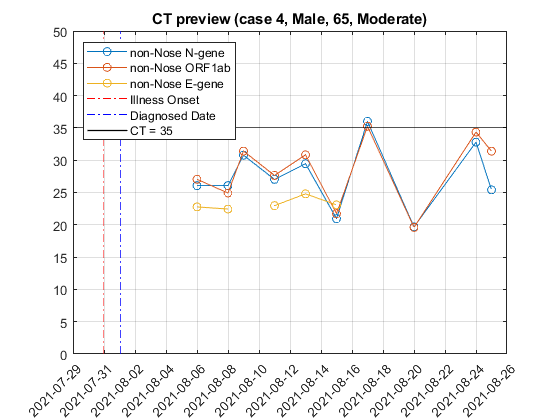


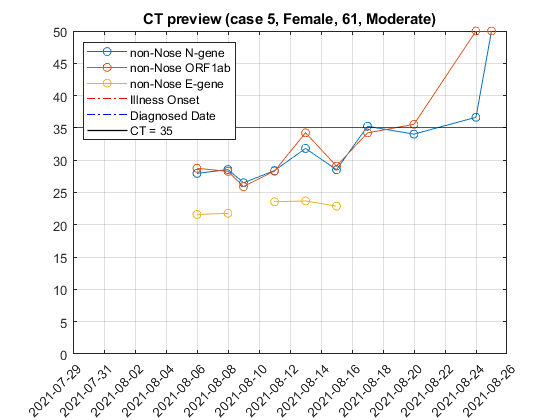


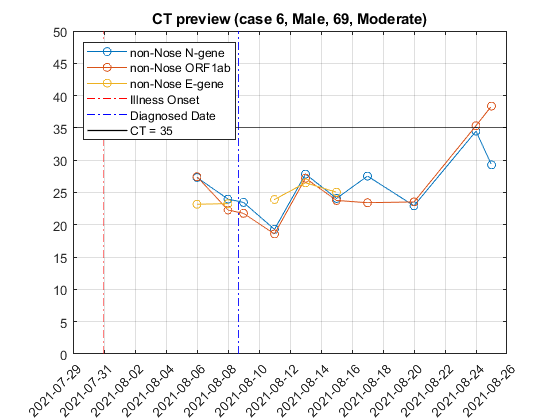


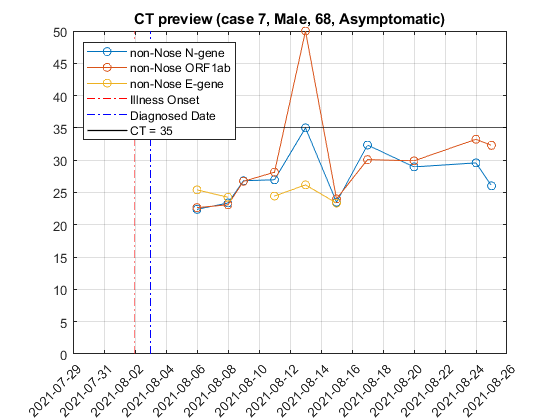


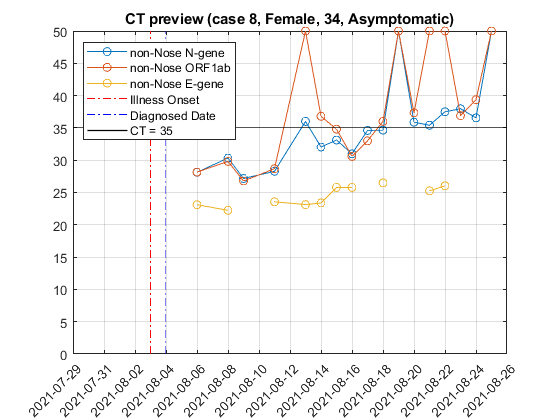


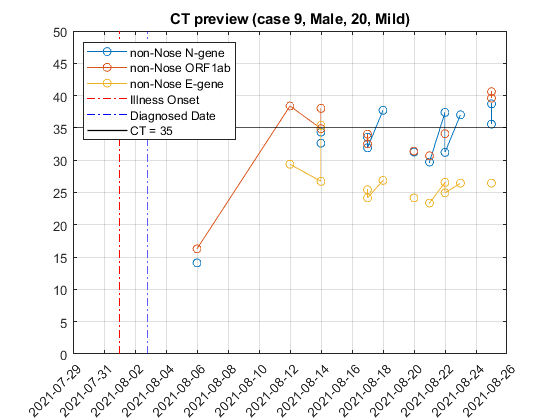


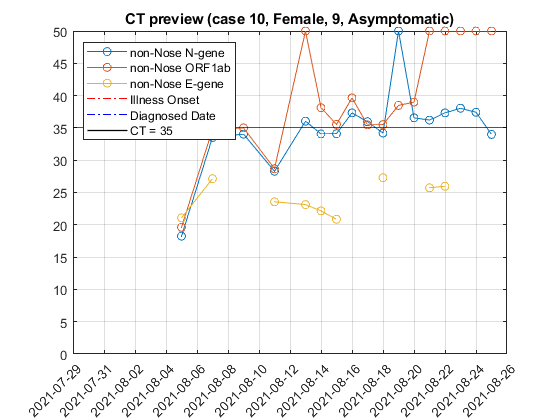


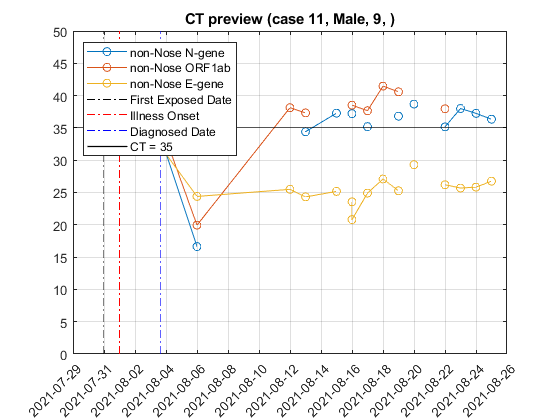


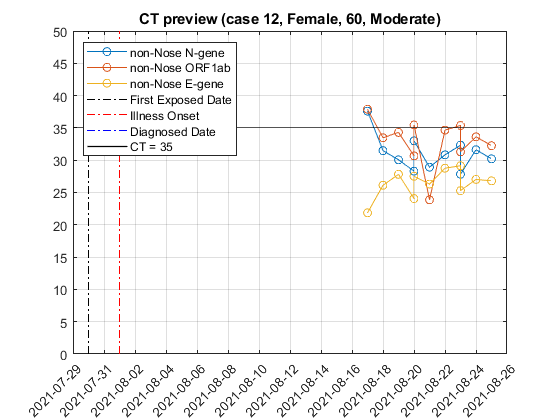


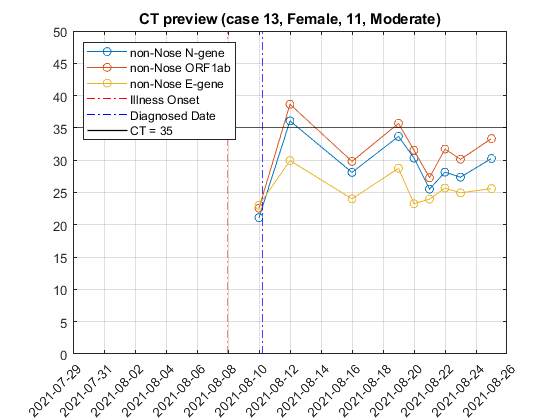


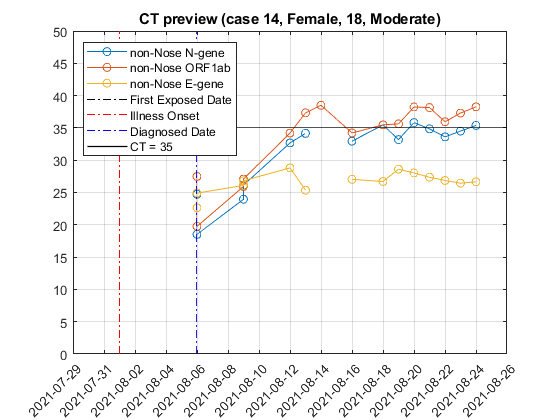


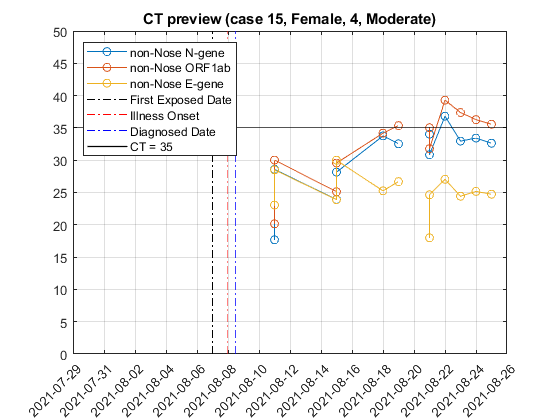


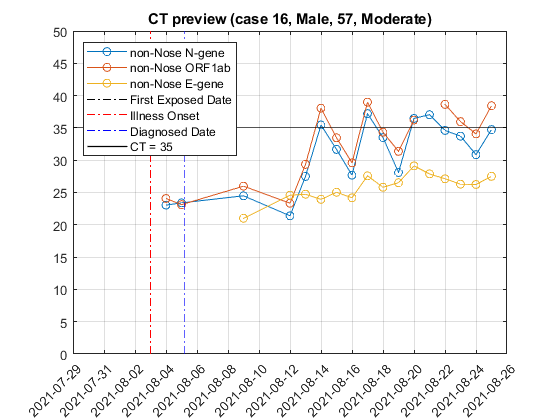


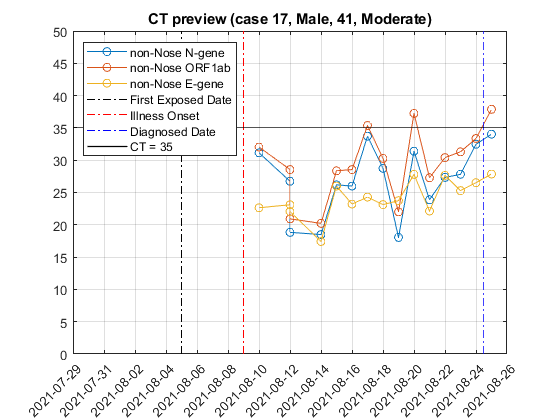


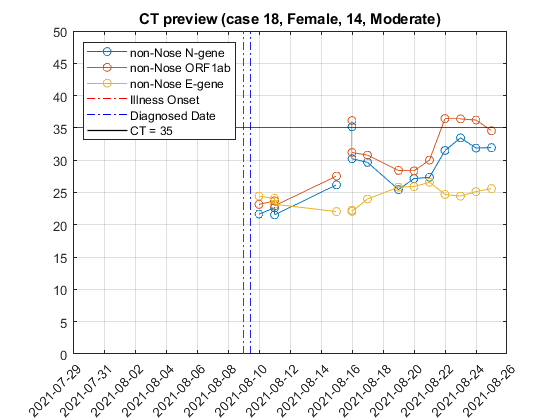


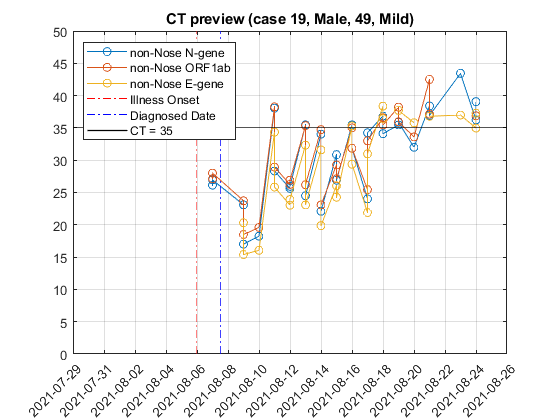


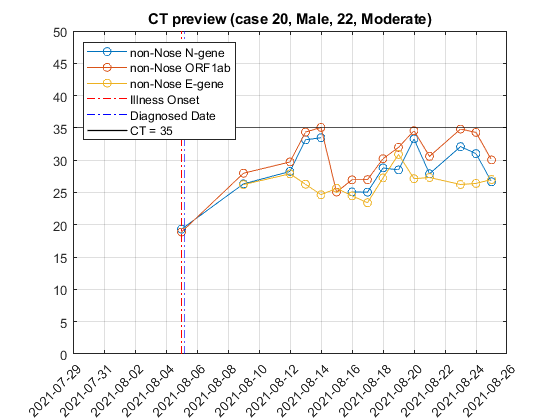


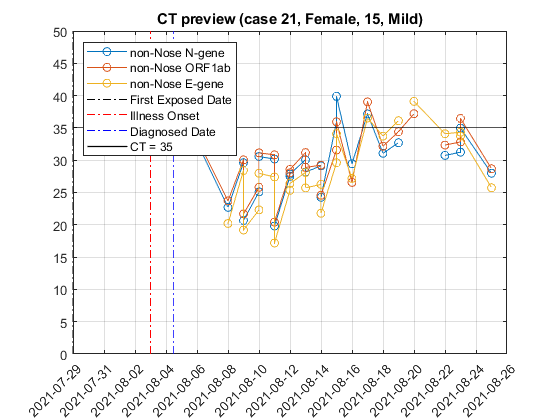


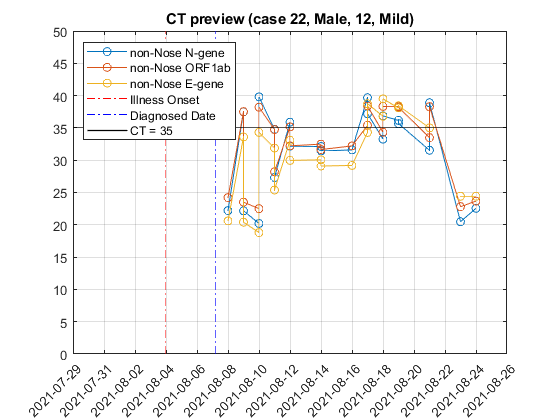


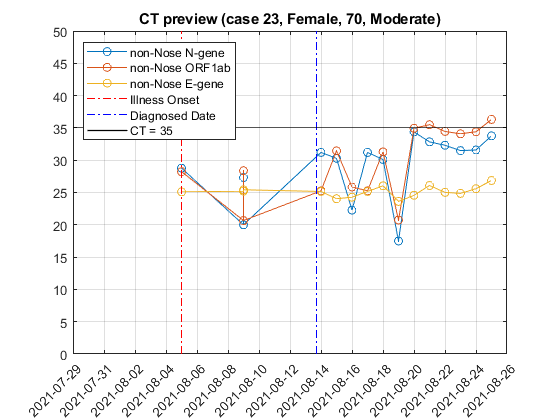


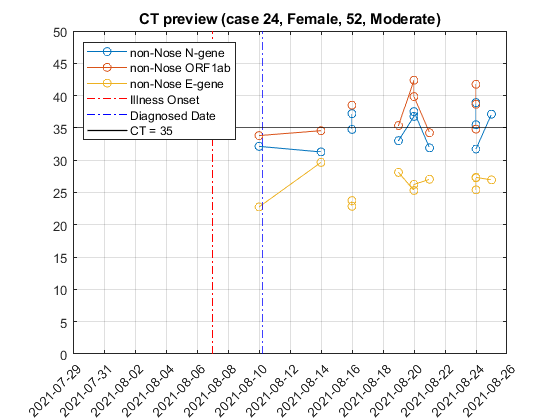


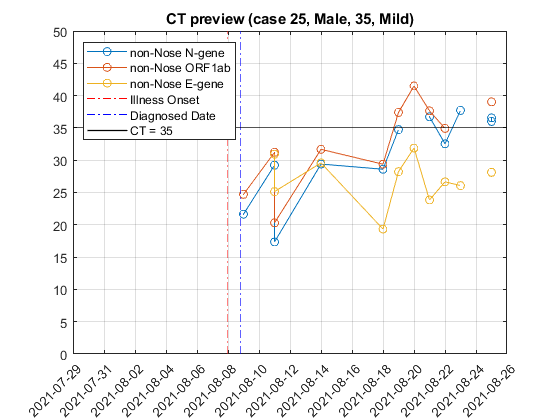


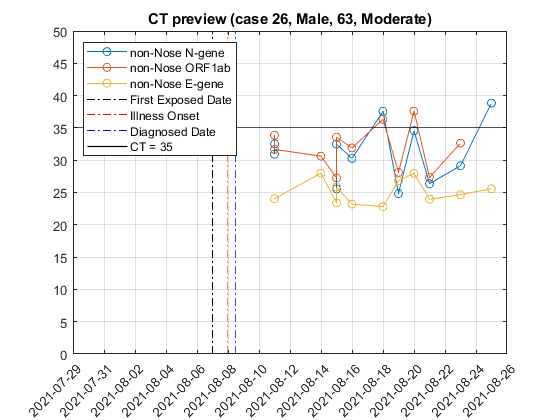


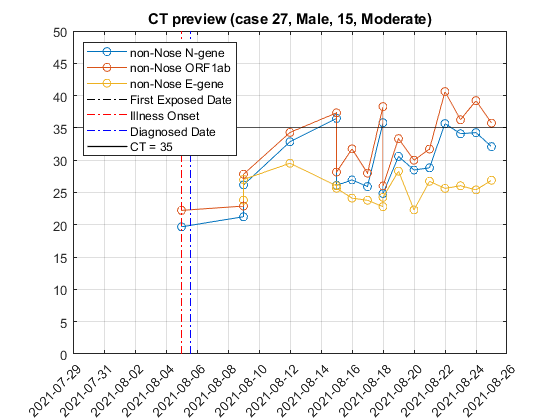


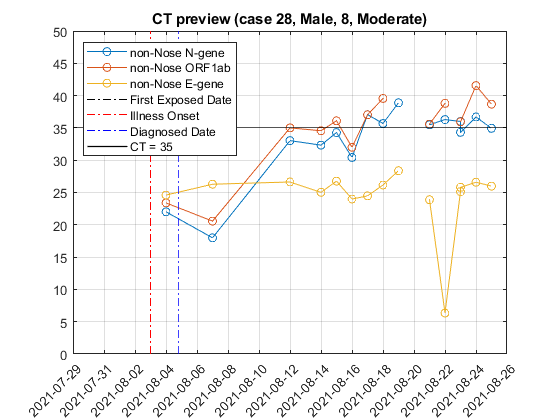


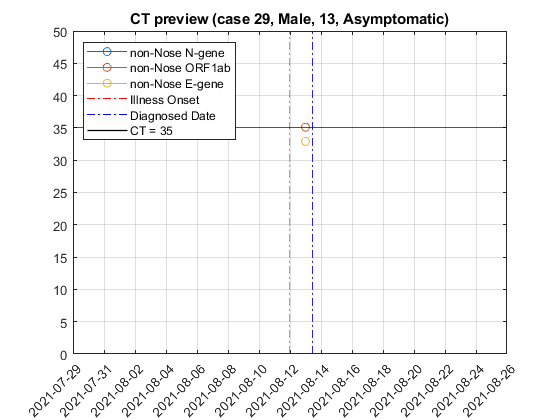


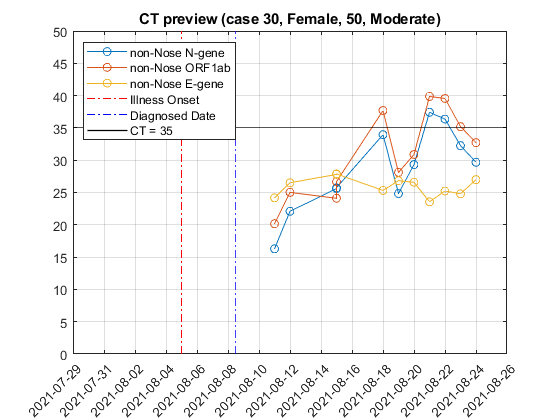


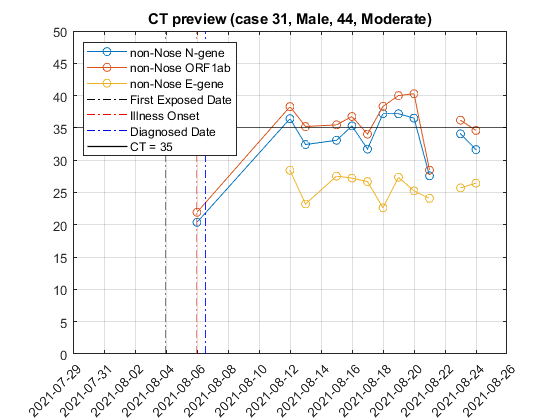


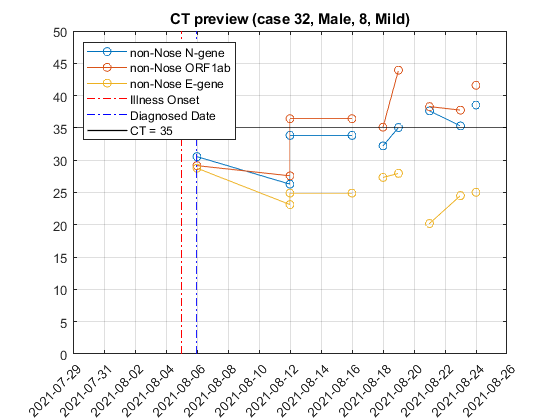


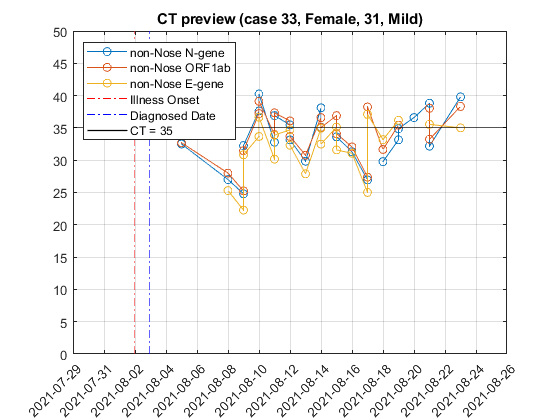


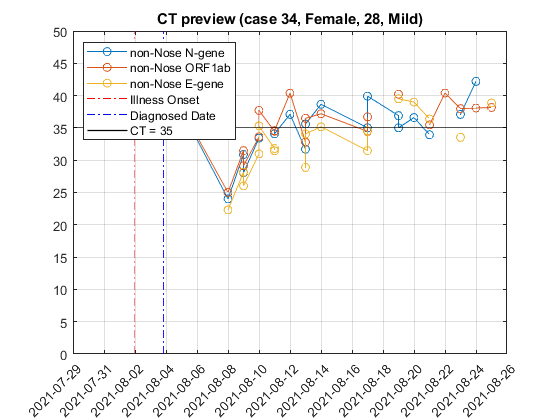


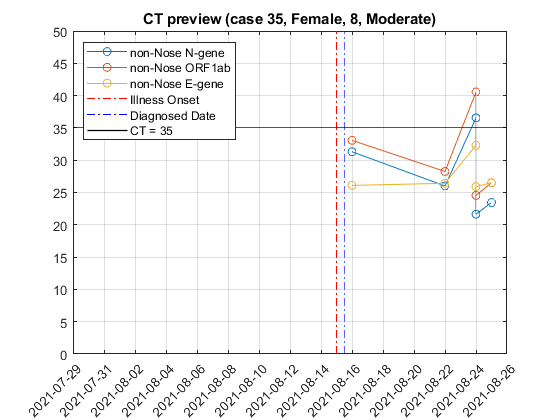


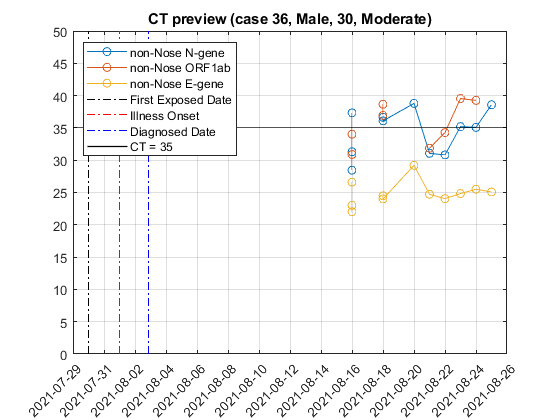


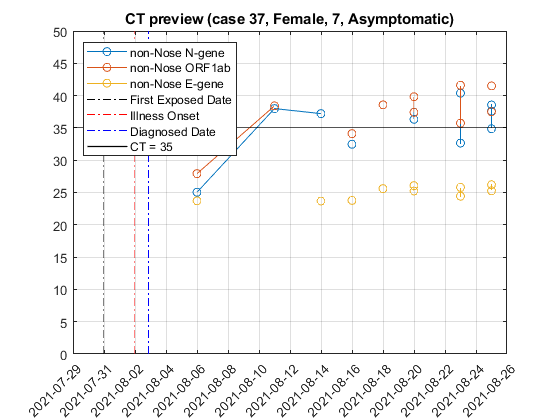


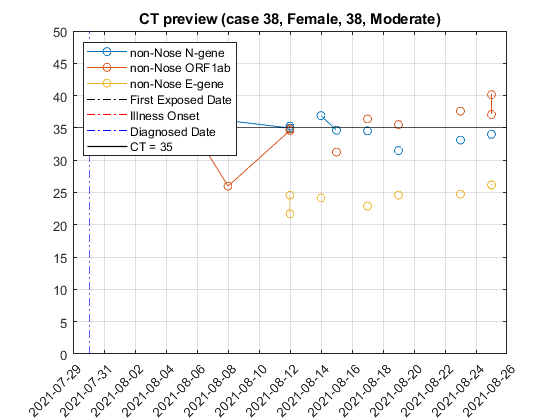


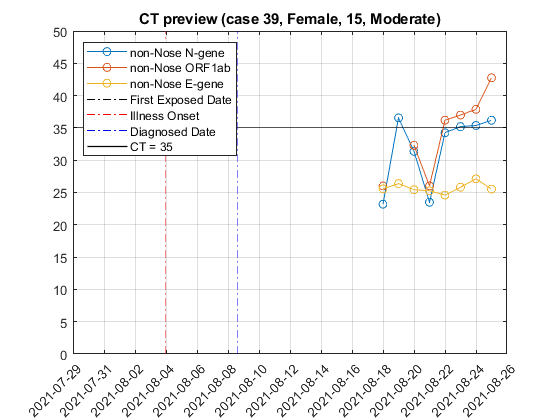


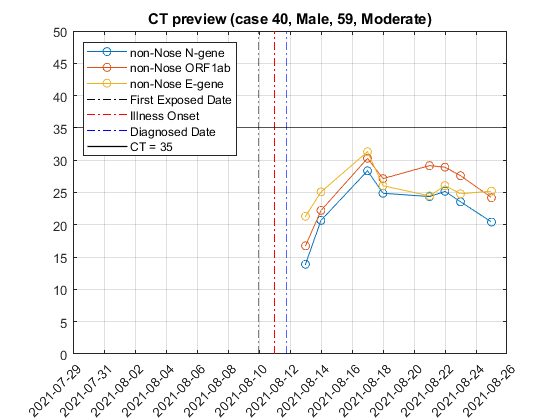


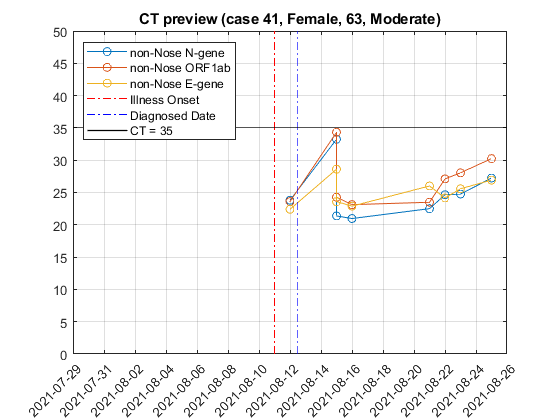


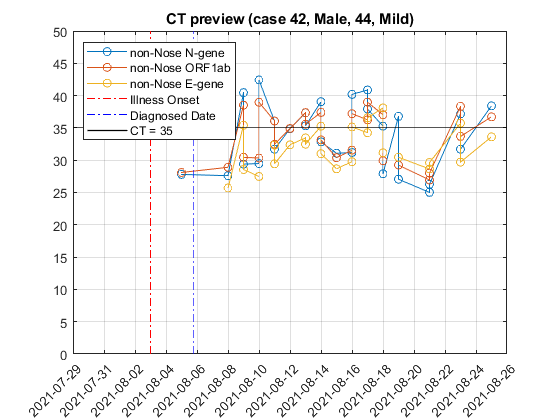


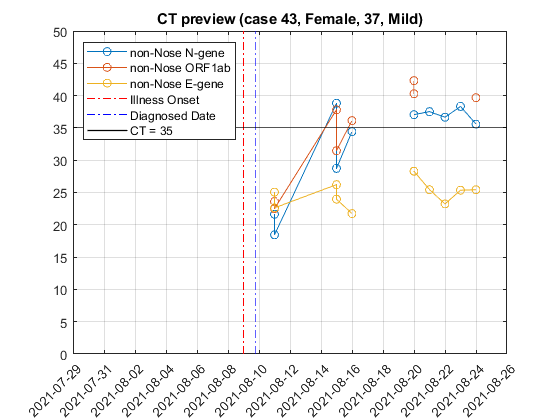


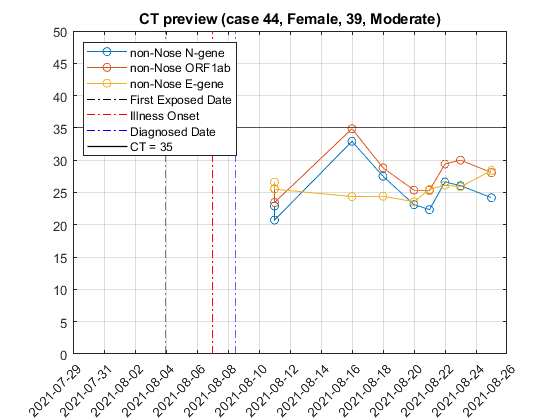


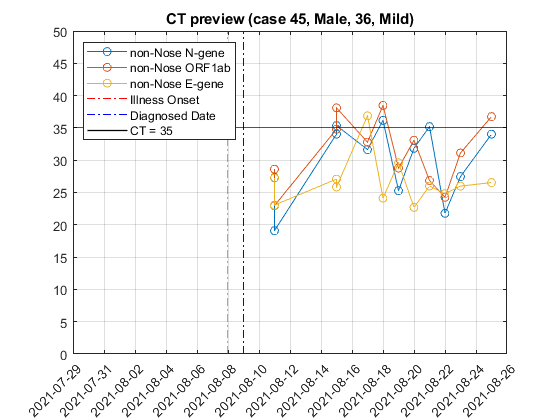


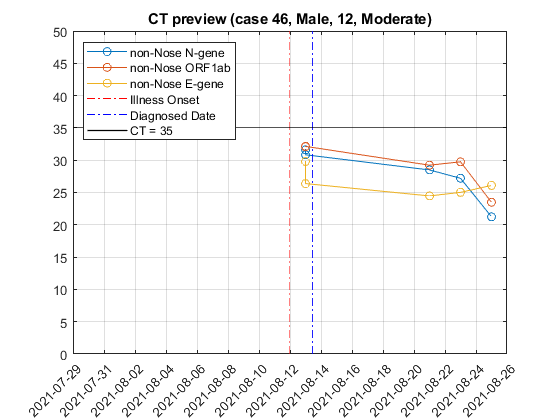


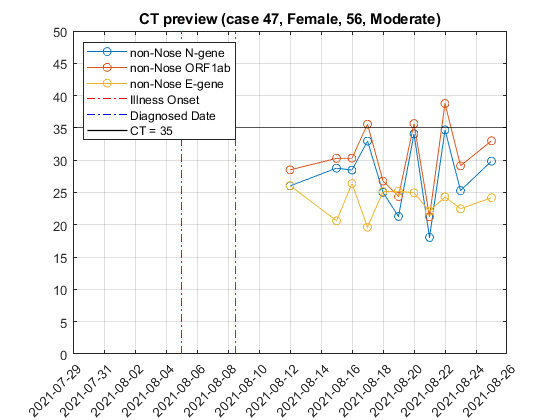


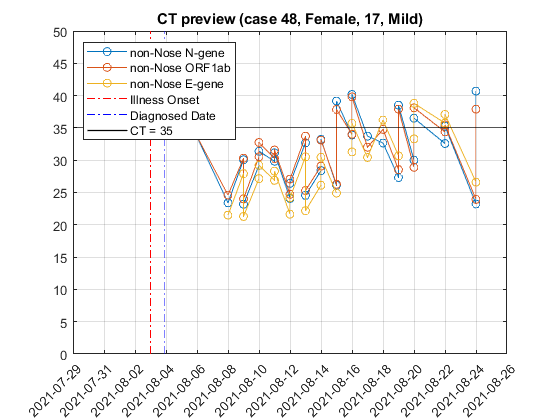


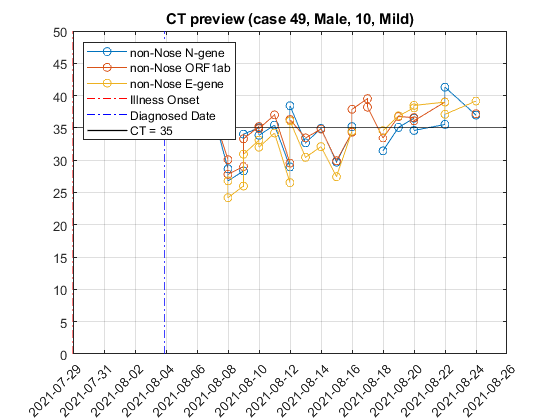


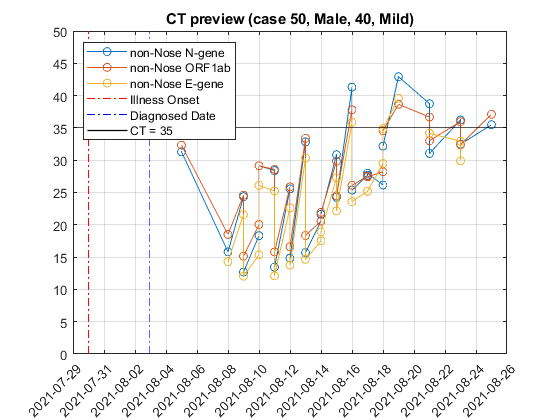


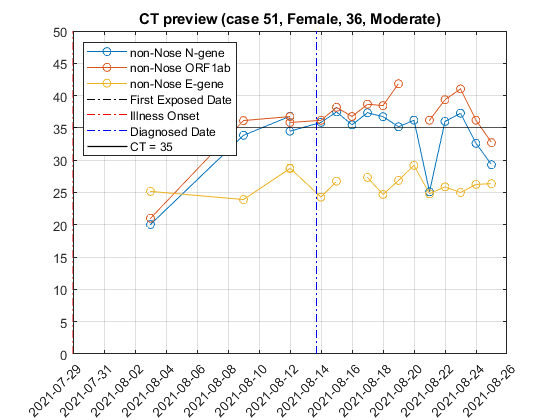


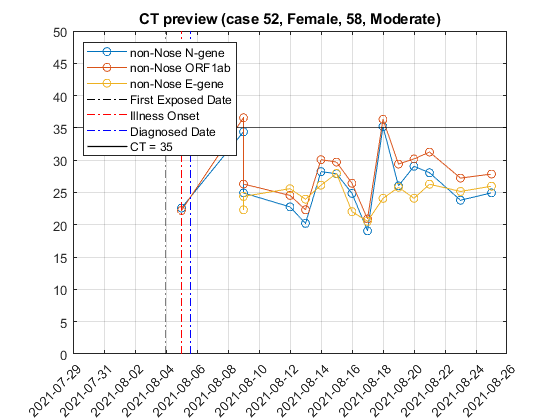


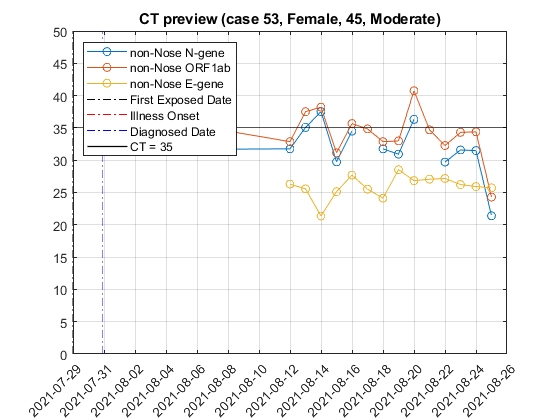


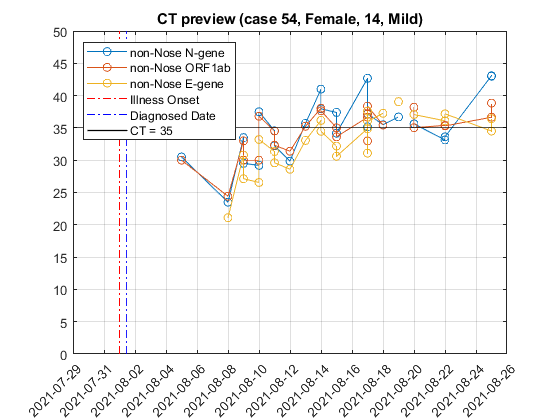


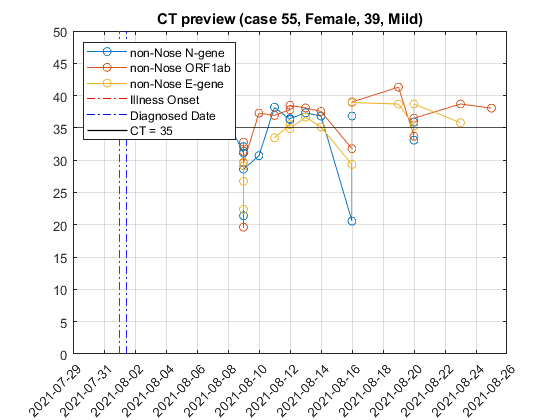


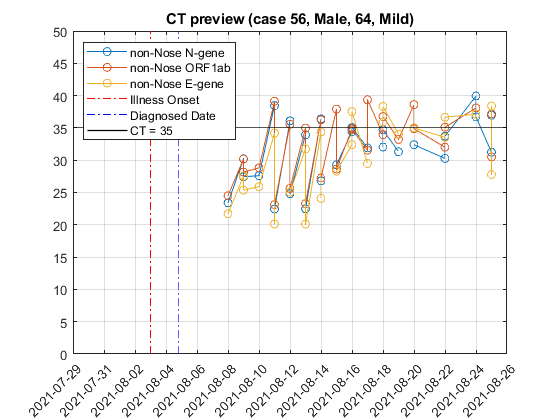


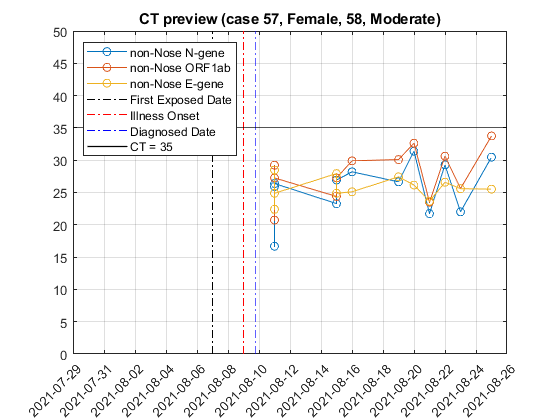


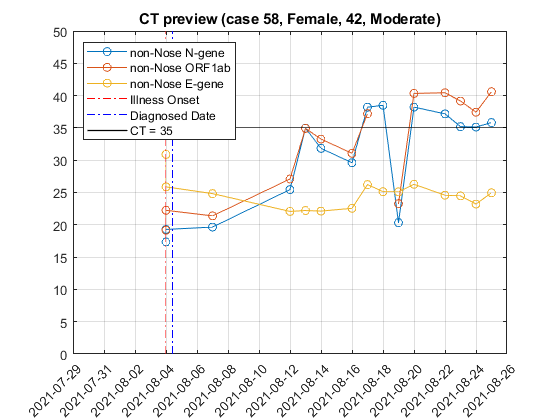


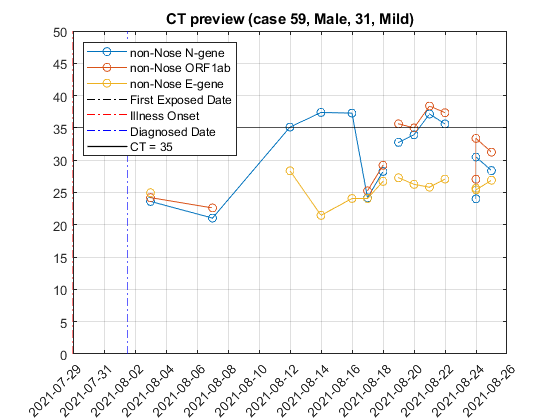


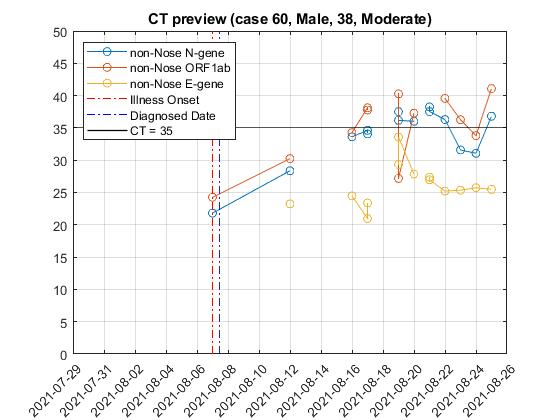


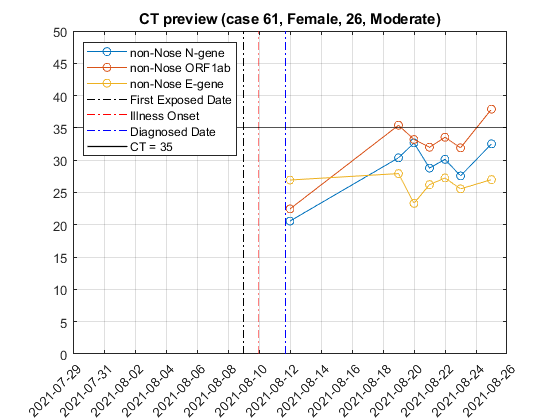


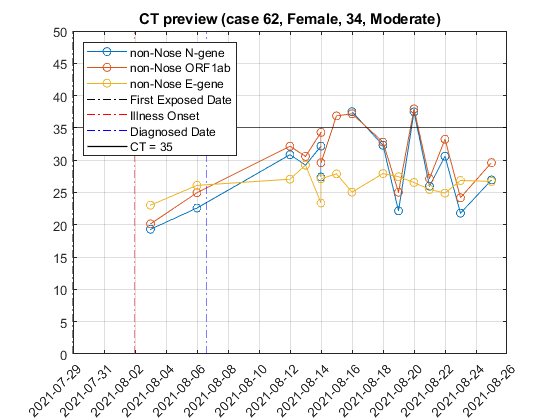


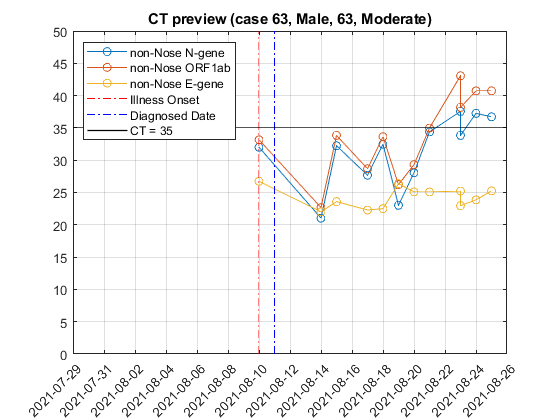


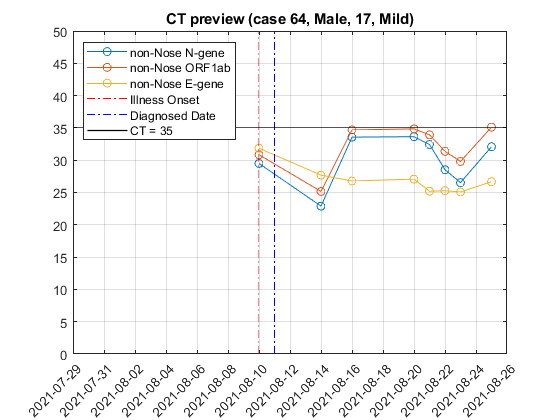


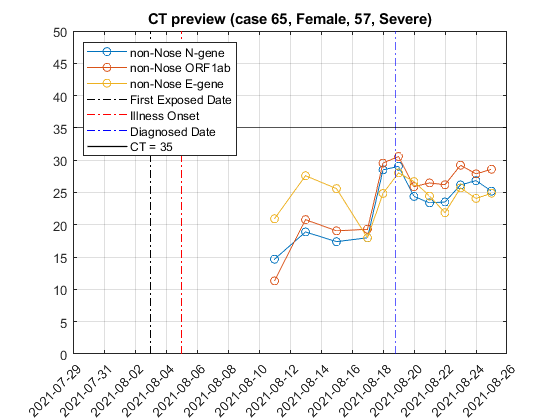


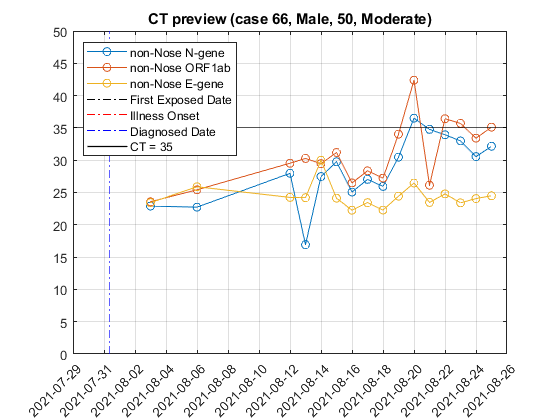


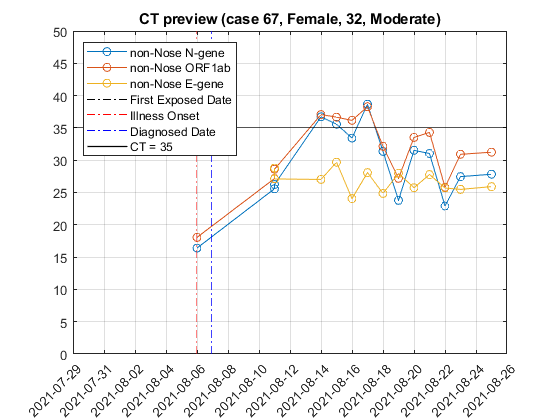


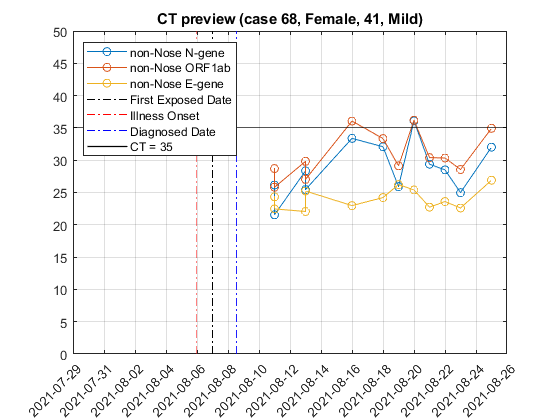


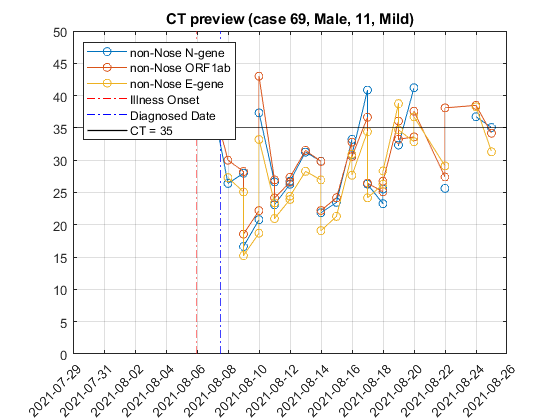


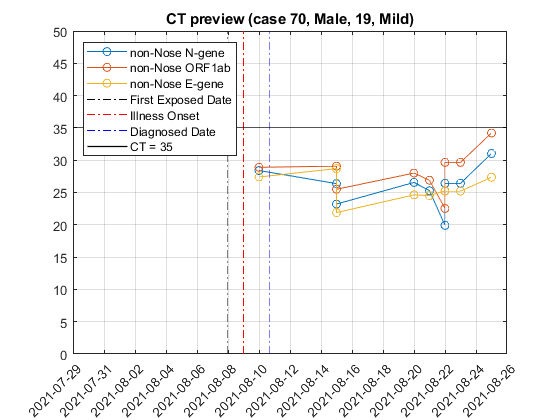


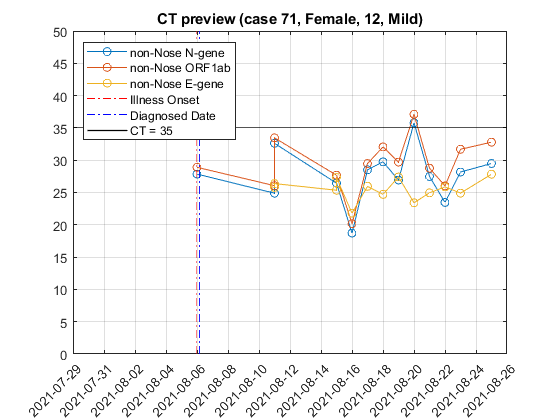


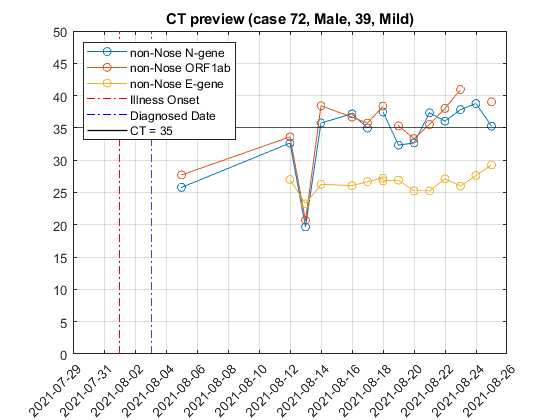


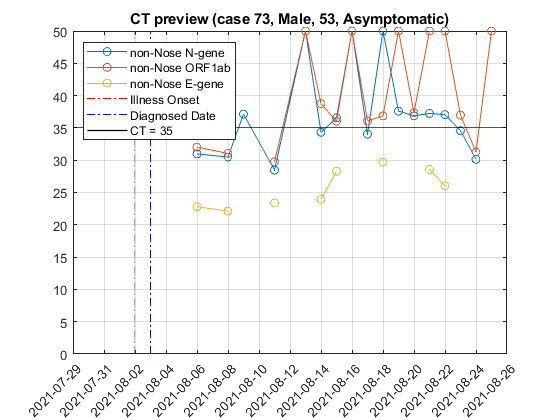


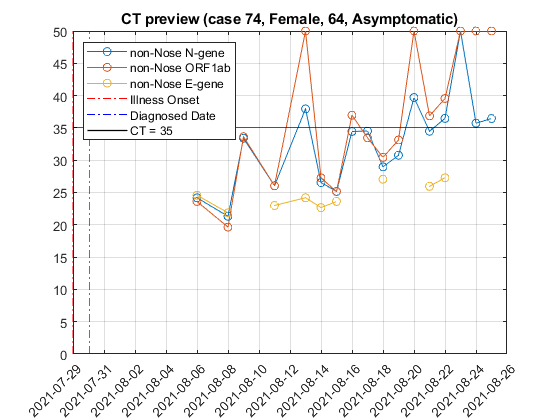


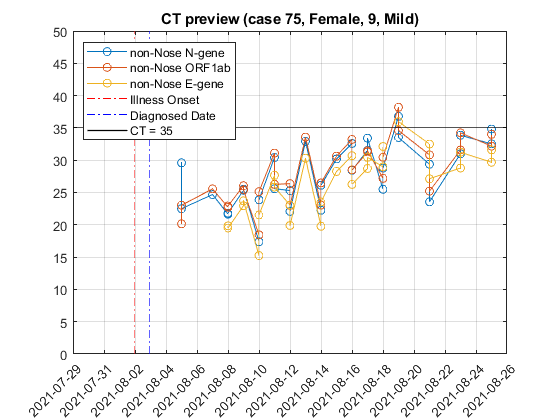


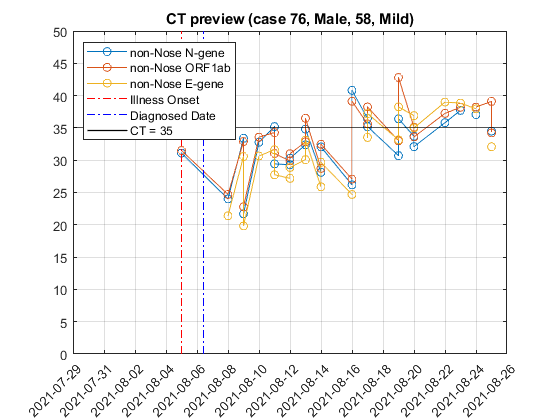


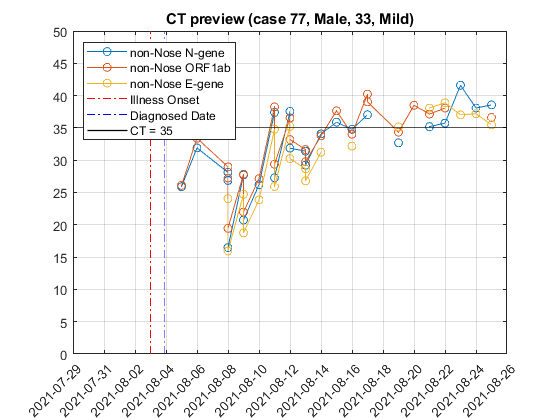


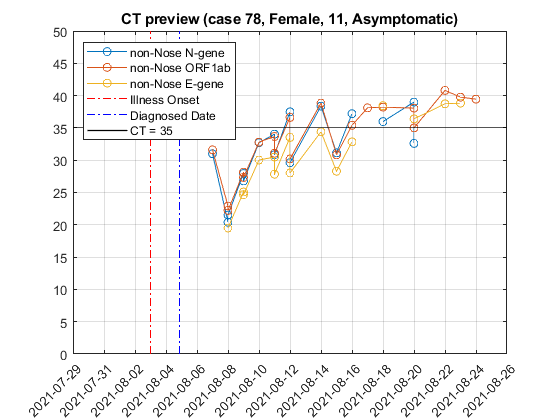


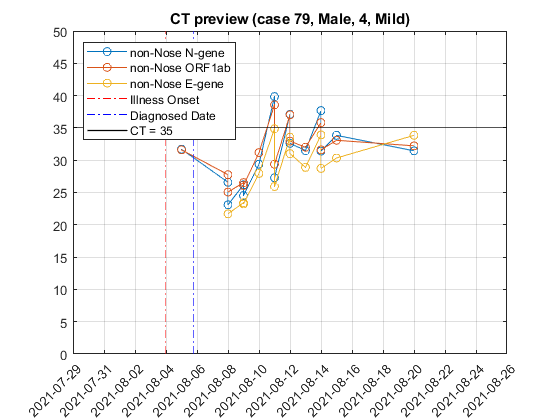


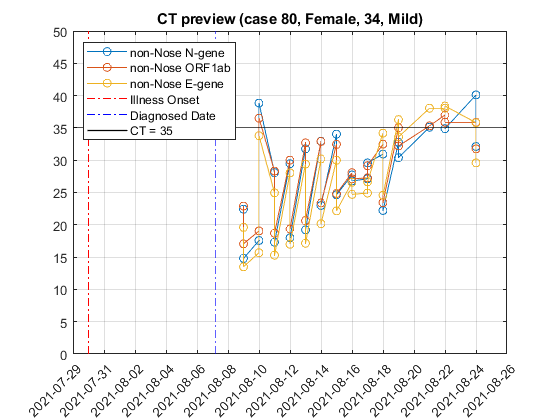


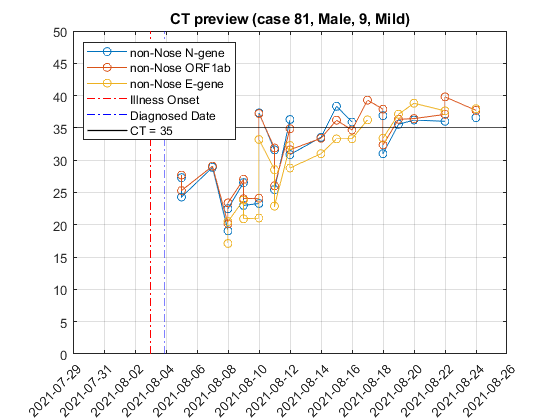


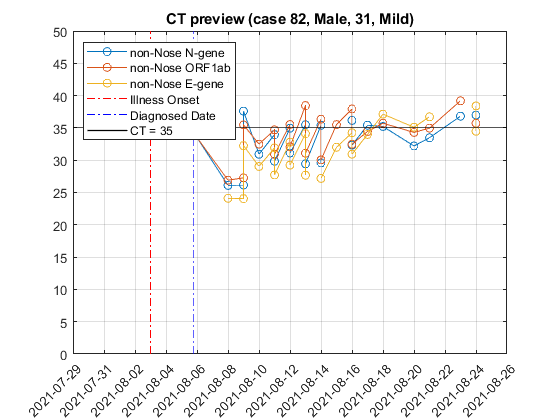


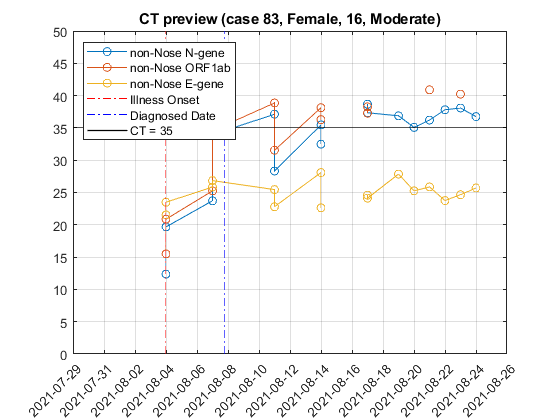


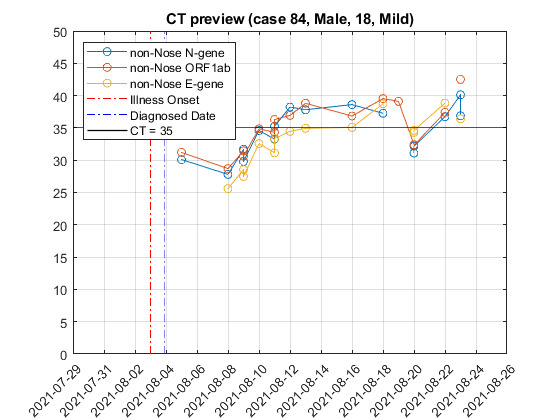


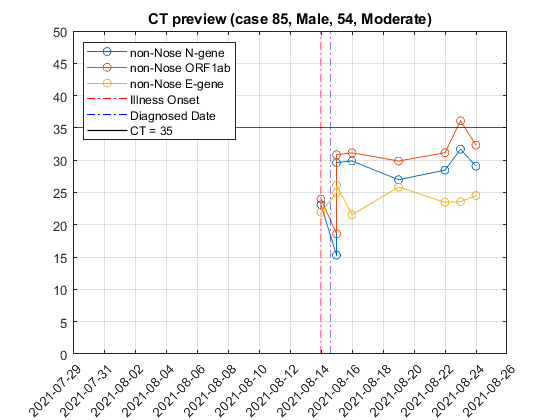


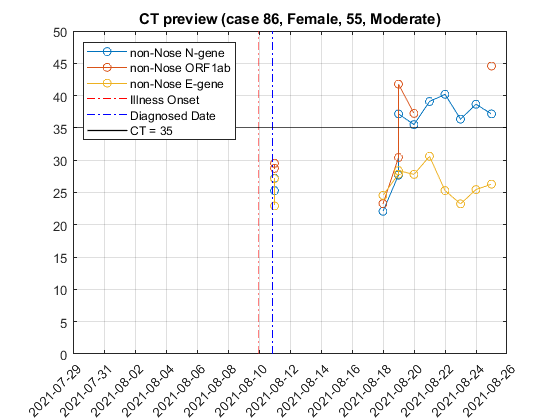


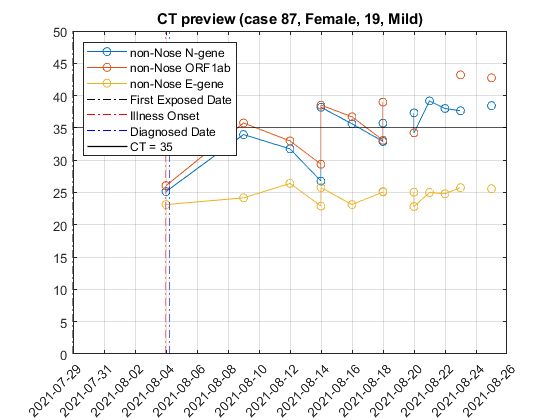


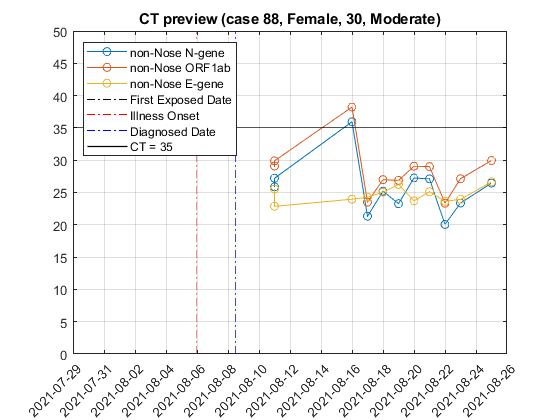


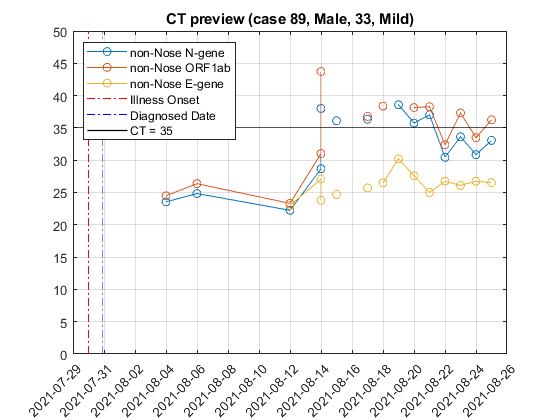


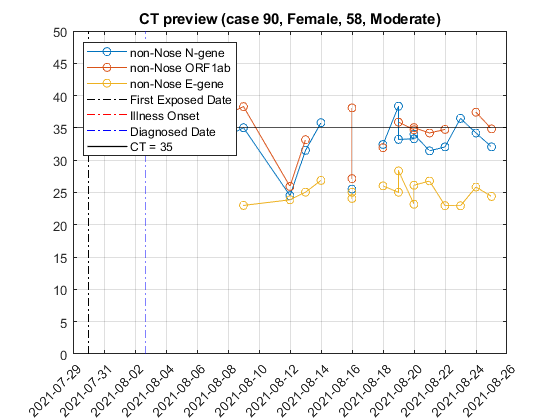


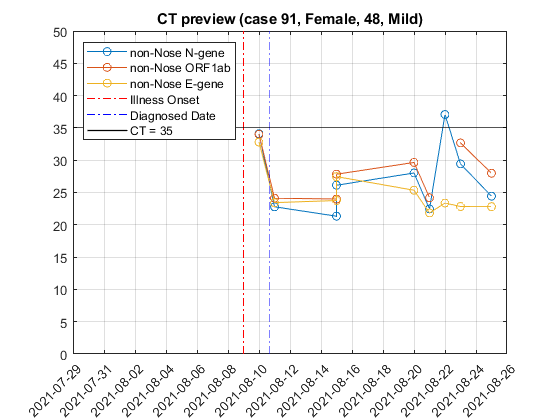


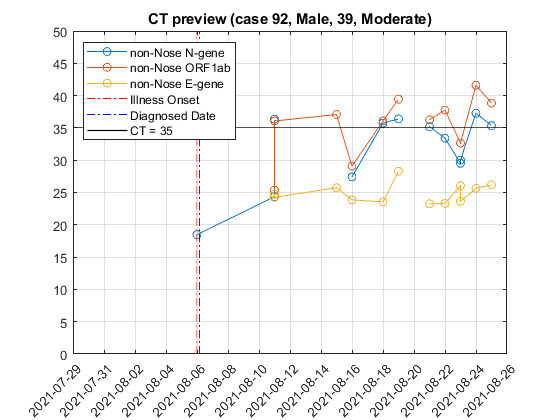


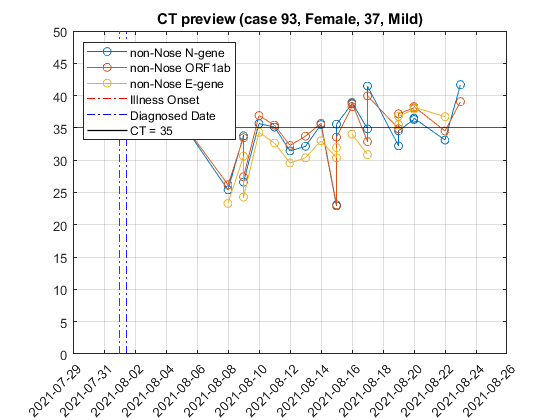


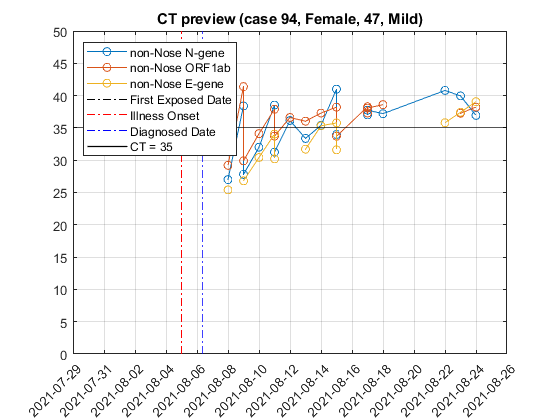


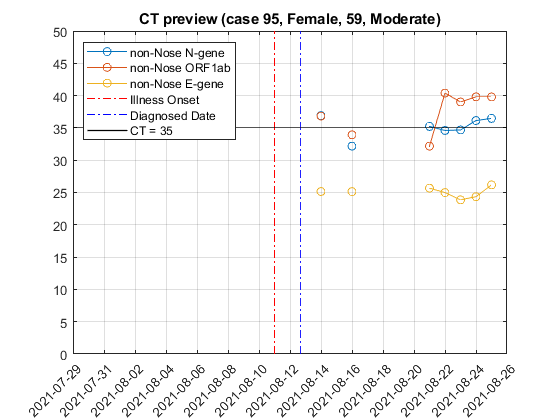


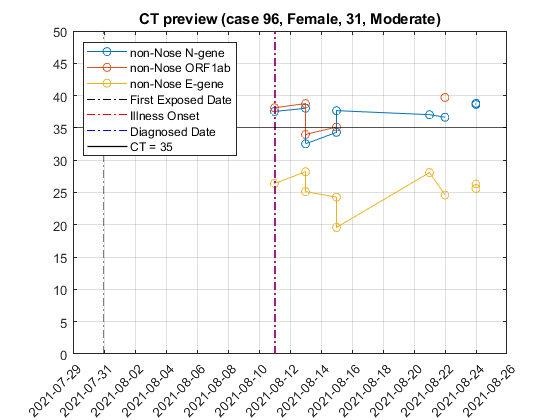


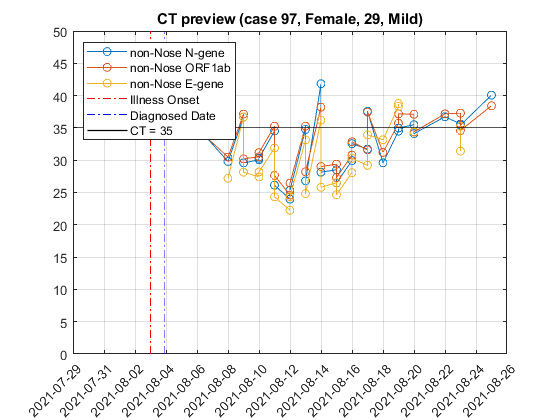


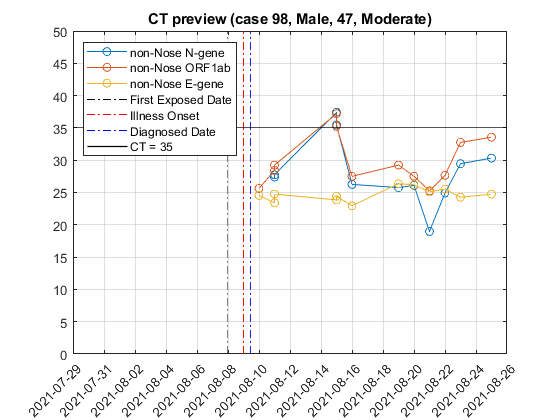


[*Published with MATLAB® R2021b*](https://www.mathworks.com/products/matlab)
